# Proton‐pumping pyrophosphatase homeolog expression is a dynamic trait in bread wheat ( *Triticum aestivum* )

**DOI:** 10.1002/pld3.354

**Published:** 2021-10-07

**Authors:** Daniel Jamie Menadue, Matteo Riboni, Ute Baumann, Rhiannon Kate Schilling, Darren Craig Plett, Stuart John Roy

**Affiliations:** ^1^ School of Agriculture, Food and Wine University of Adelaide Adelaide South Australia Australia; ^2^ Australian Centre for Plant Functional Genomics The University of Adelaide Urrbrae South Australia Australia; ^3^ Department of Primary Industries and Regions South Australian Research and Development Institute Urrbrae South Australia Australia; ^4^ Australian Plant Phenomics Facility, The Plant Accelerator The University of Adelaide Adelaide South Australia Australia; ^5^ ARC Industrial Transformation Research Hub for Wheat in a Hot and Dry Climate University of Adelaide Adelaide South Australia Australia

**Keywords:** gene expression, gene identification, homeolog, synteny analysis, vacuolar pyrophosphatases

## Abstract

Proton‐pumping pyrophosphatases (H^+^‐PPases) have been shown to enhance biomass and yield. However, to date, there has been little work towards identify genes encoding H^+^‐PPases in bread wheat ( *Triticum aestivum* ) (*TaVP*s) and limited knowledge on how the expression of these genes varies across different growth stages and tissue types. In this study, the IWGSC database was used to identify two novel *TaVP* genes, *TaVP4* and *TaVP5*, and elucidate the complete homeolog sequences of the three known *TaVP* genes, bringing the total number of bread wheat *TaVP*s from 9 to 15. Gene expression levels of each *TaVP* homeolog were assessed using quantitative real‐time PCR (qRT‐PCR) in four diverse wheat varieties in terms of phenotypic traits related to high vacuolar pyrophosphatase expression. Homeolog expression was analyzed across multiple tissue types and developmental stages. Expression levels of the *TaVP* homeologs were found to vary significantly between varieties, tissues and plant developmental stages. During early development (Z10 and Z13), expressions of *TaVP1* and *TaVP2* homeologs were higher in shoot tissue than root tissue, with both shoot and root expression increasing in later developmental stages (Z22). *TaVP2‐D* was expressed in all varieties and tissue types and was the most highly expressed homeolog at all developmental stages. Expression of the *TaVP3* homeologs was restricted to developing grain (Z75), while *TaVP4* homeolog expression was higher at Z22 than earlier developmental stages. Variation in *TaVP4B* was detected among varieties at Z22 and Z75, with Buck Atlantico (high biomass) and Scout (elite Australian cultivar) having the highest levels of expression. These findings offer a comprehensive overview of the bread wheat H^+^‐PPase family and identify variation in *TaVP* homeolog expression that will be of use to improve the growth, yield, and abiotic stress tolerance of bread wheat.

## 1 INTRODUCTION

Bread wheat (*Triticum aestivum*) is the most widely cultivated cereal in the world (FAOstat, 2019), providing 20% of the daily calorie requirements to the global population (Shiferaw et al., 2013). With the global population predicted to reach 9.1 billion by 2050, a significant increase in wheat yield is required to maintain food security (Alexandratos & Bruinsma, 2012). However, many environmental factors limit our ability to achieve the yield increases required. Wheat yields are significantly limited by increasing global temperatures, rainfall variability, and depletion of soil nutrients (Gilliham et al., 2017; Tester & Langridge, 2010). In order to reduce the impact of such abiotic stresses, wheat cultivars with greater nutrient uptake capacity and abiotic stress tolerance are required. Developing such cultivars can be assisted through the identification and characterization of beneficial genes for plant development and stress tolerance (Gilliham et al., 2017).

The inorganic vacuolar proton‐pumping pyrophosphatase (H^+^‐PPase) is a ubiquitous plant enzyme (*EC 3.6.1.1*) responsible for the breakdown of inorganic pyrophosphate (PP_i_) and the production of free energy (Baltscheffsky, 1967). Plants have two types of H^+^‐PPases, type I enzymes which require potassium (K^+^) ions for optimal function and type II enzymes that do not require K^+^ but are sensitive to cytosolic calcium (Ca^2+^) concentrations (Maeshima, 1991). Types I and II H^+^‐PPases also differ in protein localization and abundance, with type I H^+^‐PPases localized to the tonoplast and plasma membrane (Maeshima, 1991; Paez‐Valencia et al., 2011), and the type II H^+^‐PPases localized to the membrane of the Golgi apparatus (Mitsuda et al., 2001). A variety of roles for these enzymes, particularly the type I H^+^‐PPases, have been proposed (Gaxiola et al., 2016; Schilling et al., 2017), including aiding vacuolar ion sequestration (Gaxiola et al., 1999, 2001) and sugar transportation (Gaxiola et al., 2012; Khadilkar et al., 2016; Pizzio et al., 2015), regulating cytosolic PP_i_ (Ferjani et al., 2011) and assisting with nutrient uptake (Paez‐Valencia et al., 2013; Yang et al., 2007, 2014). Numerous studies have shown that transgenic plants expressing H^+^‐PPases have increased biomass and yield under saline (Bao et al., 2009; Cheng et al., 2018; Gaxiola et al., 2001; Kim et al., 2013; Li et al., 2010; Pasapula et al., 2011; Qin et al., 2013; Schilling et al., 2014; Shen et al., 2015), drought (Bao et al., 2009; Gaxiola et al., 2001; Park et al., 2005; Qin et al., 2013; Shen et al., 2015), low available nitrogen (Lv et al., 2015; Paez‐Valencia et al., 2013), and low available phosphorus (Yang et al., 2007, 2014) conditions, as well as in non‐stressed conditions (Gonzalez et al., 2010; Li et al., 2005; Pizzio et al., 2015; Schilling et al., 2014; Vercruyssen et al., 2011; Wang et al., 2014; Yang et al., 2014). Recently, it was shown that expression of Arabidopsis *AVP1* in bread wheat enhanced biomass production and yield in both field and greenhouse conditions (Regmi et al., 2020). This was in part due to enhanced remobilization of carbon from source to sink tissues (Regmi et al., 2020). While these results are very promising as a possible approach to enhance wheat yield, the limited amount of commercially approved genetically modified (GM) wheat that is currently available does not make the transgenic approach viable in the short term. An alternative approach would be to identify natural variation in the wheat homologs of *AVP1* and/or introduce new variation through genome editing or by marker‐assisted selection (MAS). Prior to this, however, it is necessary to first confirm the identity of the wheat homologs and homeologs of *AVP1*, the *TaVPs*, and their expression pattern at different developmental stages.

Type I H^+^‐PPase enzymes have been characterized in Arabidopsis (Sarafian et al., 1992) and mung bean (*V. radiata*) (Britten et al., 1989), as well as important cereal crops including barley (*H. vulgare*) (Fukuda et al., 2004; Tanaka et al., 1993), maize (*Z. mays*) (Wisniewski & Rogowsky, 2004; Yue et al., 2008), rice (*O. sativa*) (Choura & Rebai, 2005; Liu et al., 2010; Sakakibara et al., 1996), and wheat (Brini et al., 2005; Wang et al., 2009). To date, three type I H^+^‐PPase genes, *TaVP1*, *TaVP2*, and *TaVP3*, have been identified through analysis of a wheat root tissue cDNA library and the 158th GenBank expressed sequence tag (EST) database (Brini et al., 2005; Wang et al., 2009). In addition, homeologous sequences from the A, B, and D genomes have also been identified for each of the three *TaVP* genes (Wang et al., 2009); however, to our knowledge, these sequences remain unpublished. Based on semi‐quantitative gene expression analysis on one Chinese wheat variety, Wang et al. (2009) concluded that *TaVP1* homeologs were more highly expressed in root than shoot tissue, *TaVP2* homeologs were more highly expressed in shoot than root tissue, and expression of *TaVP3* homeologs was restricted to the developing seed. The publication of the wheat genome sequence (International Wheat Genome Sequencing Consortium, 2014, 2018) provides an opportunity to determine if all the wheat *TaVPs* have been identified or if there are others still awaiting discovery. Expression profiling of *TaVP* expression in multiple wheat varieties will help determine variability between different wheat accessions and help determine their role and function in the plant.

## 2 MATERIALS AND METHODS

### 2.1 Identification of wheat H^+^‐PPase homeologs

To identify homeolog specific H^+^‐PPase sequences within the wheat genome, nucleotide coding sequences (CDS) and amino acid sequences of the previously identified *TaVP1* (AY296911.1/AAP5521.1) and *TaVP2* (EU255237.1/ABX10014.1) genes, as well as the barley *HVP3* (AK362588.1/BAJ93792.1) gene, were used to query the databases of the International Wheat Genome Sequencing Consortium (IWGSC, https://www.wheatgenome.org). Sequences with >80% identity were considered as homologs, while those with identities >95% and located on the same chromosome group were considered homeologs (Wilhelm et al., 2013). Putative *TaVP* homeolog CDS were aligned in Geneious® 1.1.3 (Biomatters Ltd., Auckland, New Zealand) using the MUSCLE algorithm (default parameters), and amino acid sequences were translated, protein sequences were confirmed, and genes ID were obtained from the IWGSC RefSeq v1.1 (International Wheat Genome Sequencing Consortium, 2018). The GSDS2.0 software (Hu et al., 2015) was used to determine the intron‐exon structure of each homeolog, while protein sequence features such as secondary structures, proton binding sites, and PP_i_ binding sites were inferred using the known structure of the *V. radiata* H^+‐^PPase (BAA23649) via ESPript3 (Robert & Gouet, 2014). Protein localization was predicted from TaVP amino acid sequences using ProteinPredict (default parameters) (Yachdav et al., 2014).

### 2.2 Sequence alignment and phylogenetic analysis of H^+^‐PPase proteins

Type 1 Plant H^+^‐PPase protein sequences were aligned in Jalview (Waterhouse et al., 2009) using the MUSCLE algorithm (default settings). The resulting alignment was used for molecular phylogenetic analysis in MEGA 6® (Tamura et al., 2013) and formatted with iTOL v3 (Letunic & Bork, 2016). Evolutionary history was calculated using the Maximum Likelihood methodology (Mount, 2008) and validated with 1,000 bootstrap replicates.

### 2.3 Synteny analysis

Synteny analysis was performed using the 24 genes flanking the *TaVP*s on genome A as the search query in the BLASTP function of Geneious Prime® 2020 software. The query was searched against public available genomes of wheat (*Triticum_aestivum*, IWGSC), barley (*Hordeum_vulgare*, IBSC_v2), rice (*Oryza_sativa*, IRGSP‐1.0), maize (*Zea_mays*, B73_v4), Arabidopsis (*A. thaliana* TAIR10), and mung bean (*Vigna_radiata*, Vradiata_ver6). Genes with *P* value below 10^−90^ were considered as orthologs. Gene IDs were confirmed at the EnsemblPlants orthologues database (https://plants.ensembl.org/). All orthologs found had a *P* value below 1.24 × 10^−92^ (Table S1).

### 2.4 Plant growth conditions for *TaVP* expression analysis in bread wheat

To assess *TaVP* homeolog expression through time, four wheat varieties were selected based on commonly observed phenotypes of *AVP1* over‐expressing plants: high biomass, high yield, and improved seedling vigor. Uniform size seed from Vigour18 (early seedling vigor, CSIRO), Mocho de Espiga Branca (high biomass landrace, Portugal), Scout (elite Australian cultivar, LongReach Plant Breeding), and Buck Atlantico (high biomass cultivar, Argentina) were UV sterilized for 5 min and germinated on moist paper towel in petri dishes for 6 days in the greenhouse. Seedlings were transplanted into PVC tubes (40 mm × 280 mm) containing polycarbonate plastic beads (Plastics Granulated Services, Adelaide, Australia) and placed into a flood‐drain hydroponics system (Shavrukov et al., 2012) containing .2 mM NH_4_NO_3_, 5.0 mM KNO_3_, 2.0 mM Ca (NO_3_)_2_, 2.0 mM MgSO_4_, .1 mM KH_2_PO_4_, .5 mM Na_2_Si_3_O_7_, 50 μM NaFe_(III)_EDTA, 5 μM MnCl_2_, 10 μM ZnSO_4_, .5 μM CuSO_4_, .1 μM Na_2_MoO_4_, and rain water to a total of 80 L (Shavrukov et al., 2012). Plants were grown in a randomized block design in a greenhouse at The Plant Accelerator® (Urrbrae, South Australia; latitude: −34.971353, longitude: 138.639933) between April 20 and May 25, 2016. The greenhouse maintained a 22°C/15°C day/night temperature regime and 60–80% humidity. Nutrient solutions were changed every 9 days, and pH was maintained between 6.5 and 7.0 by adjusting with 3.2% HCl. Tissues were collected from six replicates of each variety as follows: total shoot and total root from seedlings prior to transplantation (Z10); third leaf, root, and third leaf sheath tissue from plants with fully expanded third leaves (Z13); and third leaf, root, and third leaf sheath tissue, as well as the first fully expanded leaf from the first and second tillers (Z22). Samples were collected once the required developmental stage was reached for each variety. For the final developmental stage (Z75), developing grain samples were collected between October 14 and 27, 2015, from the 2015 ACPFG Wheat Diversity field trial at Tarlee, South Australia (latitude: −34.282156, longitude: 138.772988). Plants were grown in 5 m × 1.65 m plots, on a sodosol soil (pH [CaCl_2_] = 5.98 in top 10 cm, increasing to 7.1 further down the profile) using standard agronomic practices for the region—crops were rainfed with fertilizer application applied at sowing (100 L ha^−1^ Granulock Z 25 (Incitec Pivot Limited, Australia) (N:P:K:Zn – 25:13:00:.6), and further applications of nitrogen, in the form of 50 and 75 L ha^−1^ Easy N (Incitic Pivot Limited), at third leaf and flag leaf emergence, respectively). Five samples were collected from each wheat variety grown in two replicate plots, with each sample consisting of four individual grains from the central florets. All developmental stages were assessed according to the Zadoks growth scale (Zadoks et al., 1974).

### 2.5 RNA extraction and cDNA synthesis

RNA was extracted from tissue samples collected from greenhouse grown plants using a DirectZol RNA purification kit (Zymo Research, Irvine, United States), with contaminating DNA removed using an in‐column DNase treatment (Zymo Research). Due to low RNA yield from grain samples using the DirectZol methodology, likely due to high starch content within developing wheat grains, a phenol‐chloroform extraction (Chomczynski, 1993) was used to purify RNA from grain samples. Contaminating DNA was removed from the grain RNA samples using an Ambion DNA‐free treatment (Invitrogen, Carlsbad, United States). RNA concentrations were standardized to 500 ng using a NanoDrop 1000 spectrophotometer (Thermo Fisher Scientific, Waltham, United States), and cDNA was synthesized using an AffinityScript cDNA synthesis kit (Agilent Technologies, Santa Clara, United States) according to the manufacturer's instructions.

### 2.6 Expression analysis of *TaVP* homeologs via quantitative real‐time PCR (qRT‐PCR)

Homeolog specific primers were designed with Primer3 (version 4..0) (Table S1) and the specificity of each primer pair was verified by melt curve analysis (Fletcher, 2014) (Figure S1) and Sanger sequencing. Although 10 primer sets were designed to amplify the *TaVP4‐D* homeolog, only one was found to be specific. While this primer set successfully amplified *TaVP4‐D* from gDNA and cDNA samples (pooled from various tissue types of the four wheat varieties) via standard PCR, this primer set was not suitable for qPCR (Figure S1). As such, expression data for the *TaVP4‐D* homeolog were not able to be obtained. qPCR was performed with KAPA SYBR® Fast qRT‐PCR kit Master Mix (Kapa Biosystems, Wilmington, United States), and amplification was monitored in real‐time on a QuantStudio™ 6 Flex Real‐Time PCR System (Applied Biosystems, Foster City, United States). Reference gene stability was assessed with the geNorm function of qBASE+ software using default settings (Hellemans et al., 2007). As the expression levels of the reference genes varied between the developmental stages, expression values within each growth stage were normalized relative to the two most stable reference genes. Z10 and Z13 data were normalized against *TaActin* (AY663392) and *TaGAPDH* (EU022331), Z22 data were normalized against *TaEFA2* (M90077) and *TaCyclophilin* (AY456122), and Z75 data were normalized against *TaGAPDH* and *TaEFA2*. Gene expression relative to the control genes (normalized relative quantity, NRQ) was calculated using equations from Hellemans et al. (2007). A heat map of the expression data was generated with log transformed data using the Morpheus software created by the Broad Institute (https://software.broadinstitute.org/morpheus/). Gene expression data were statistically analyzed in GenStat® version 15.3 (two‐way ANOVA, *P* ≤ .05, Tukey's 95% confidence interval).

## 3 RESULTS

### 3.1 Fifteen H^+^‐PPase genes are present in bread wheat

Analysis of the genomic database of the International Wheat Genome Sequencing Consortium (IWGSC, https://www.wheatgenome.org) (Caugant, 2016) identified five H^+^‐PPase genes (Table 1). Three of these genes had previously been identified (*TaVP1*, *TaVP2*, and *TaVP3*), while two were novel genes (*TaVP4* and *TaVP5*). Homeologous sequences from the A, B, and D genomes were identified for each of the five *TaVP* genes, bringing the total number of H^+^‐PPase genes within the bread wheat genome to 15 (Table 1). Homeologous sequences of each gene shared >98% sequence identity. *TaVP1* and *TaVP2* homeologs are located on the short and long arms of chromosome 7, respectively. The TaVP1 protein sequences had 96–98% sequence identity to the TaVP1 consensus sequence used to query the IWGSC assembly, while the TaVP2 sequences had 85% identity (Table 1). The *TaVP3* and *TaVP4* homeologs are located on the short and long arms of chromosome 1, respectively, and showed lower sequence identity to the TaVP1 query sequence (73–75%). Amino acid sequences of the *TaVP5* homeologs had the lowest sequence identity to the TaVP1 query sequence (36%). *TaVP5A* is not allocated to a specific chromosome in the Ref 1.1 assembly, while *TaVP5‐B* and *TaVP5‐D* are located on the long arm of chromosome 6B and 6D (Table 1).

**TABLE 1:** Details of the 15 *TaVP* homeologs identified in the bread wheat genome

Gene	ID	Chr	CDS (bp)	Protein (AA)	Identity to *TaVP1* (%)	Predicted protein localization and confidence level (%)
*TaVP1‐A*	TraesCS7A02G141300.2	7AS	2,238	745	96.2	Vacuole membrane (99)
*TaVP1‐B*	TraesCS7B02G042600.1	7BS	2,289	762	97.8	Vacuole membrane (99)
*TaVP1‐D*	TraesCS7D02G142600.2	7DS	2,286	761	97.8	Vacuole membrane (98)
*TaVP2‐A*	TraesCS7A02G517700.2	7AL	2,328	775	84.8	Vacuole membrane (84)
*TaVP2‐B*	TraesCS7B02G433800.1	7BL	2,331	776	85.1	Vacuole membrane (84)
*TaVP2‐D*	TraesCS7D02G507600.1	7DL	2,328	775	84.8	Vacuole membrane (83)
*TaVP3‐A*	TraesCS1A02G107200.1	1AS	2,298	765	73.4	Vacuole membrane (69)
*TaVP3‐B*	TraesCS1B02G124600.1	1BS	2,295	764	73.3	Vacuole membrane (69)
*TaVP3‐D*	TraesCS1D02G107600.1	1DS	2,298	765	73.5	Vacuole membrane (68)
*TaVP4‐A*	TraesCS1A02G253600.1	1AL	2,322	773	74.7	Vacuole membrane (72)
*TaVP4‐B*	TraesCS1B02G264600.	1BL	2,328	775	74.2	Vacuole membrane (72)
*TaVP4‐D*	TraesCS1D02G253200.1	1DL	2,328	775	74.2	Vacuole membrane (72)
*TaVP5‐A*	TraesCSU02G080600.1	Un	2,400	799	35.8	Golgi membrane (81)
*TaVP5‐B*	TraesCS6B02G376400.1	6BL	2,400	799	35.9	Golgi membrane (81)
*TaVP5‐D*	TraesCS6D02G326300.1	6DL	2,400	799	35.7	Golgi membrane (81)

*Note*: Gene ID, coding sequence (CDS) and protein lengths, sequence identity to the *TaVP1* consensus sequence (AAP55210.1) used to query the IWGSC databases and predicted protein localization and confidence level obtained with ProteinPredict are shown for each homeolog.

Abbreviation: Un, unknown.

**FIGURE 1:**
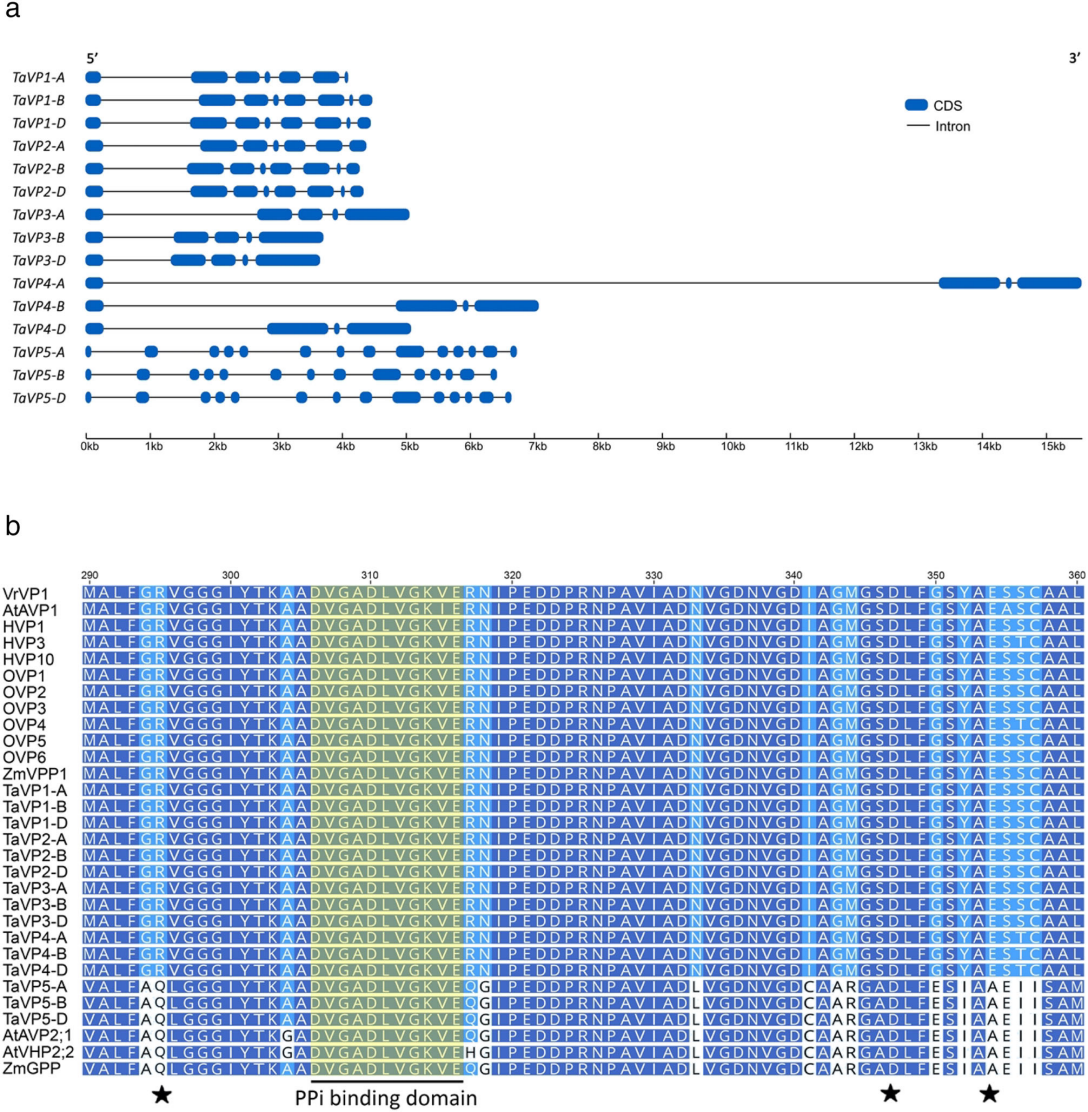
Intron‐exon analysis and amino acid alignment of *TaVP* homeologs. (a) Intron‐exon structures of *TaVP* wheat homeologs. Exons and introns are represented by blue and gray lines, respectively. Scale bars are indicated for both, with sequences shown in a 5′ to 3′ direction. (b) Amino acid sequence alignment of a highly conserved region (amino acid residues 285–355) containing a pyrophosphatase binding domain and three residues involved in proton translocation (indicated by stars). Alignment contains the TaVP homeologs and type I orthologs from *Vigna radiata* (VrVP1), *Arabidopsis thaliana* (AtAVP1), *Hordeum vulgare* (HVP1, HVP3, HVP10), *Zea mays* (ZmVPP1), and *Oryza sativa* (OVP1–6), as well as type II orthologs of *Arabidopsis thaliana* (AtAVP2;1, AtVHP2;2) and *Zea mays* (ZmGPP). Shading indicates conservation level of amino acid residues where dark blue = 100%, light blue = 80–99%, purple = 60–79%, and no color = < 60%

Exon prediction analysis confirmed the public annotations from the IWGSC RefSeq v1.1, *TaVP2‐B*, *TaVP2‐D*, and all *TaVP1* homeologs contain 8 exon regions, while the *TaVP2‐A* homeolog contained 7 (Figure 1a). *TaVP3* homeologs contained 5 exons while *TaVP4* homeologs contained 4 exons, and *TaVP5* homeologs contained 14 exons (Figure 1a). The length of the first introns in *TaVP4* homeologs were considerably larger than the other *TaVP* sequences, which averaged 1.5 kb (Figure 1a). The first intron of *TaVP4‐A* was approximately 12 kb, while the first introns of *TaVP4‐B* and *TaVP4‐D* were approximately 5 and 2.5 kb, respectively (Figure 1a).

**FIGURE 2:**
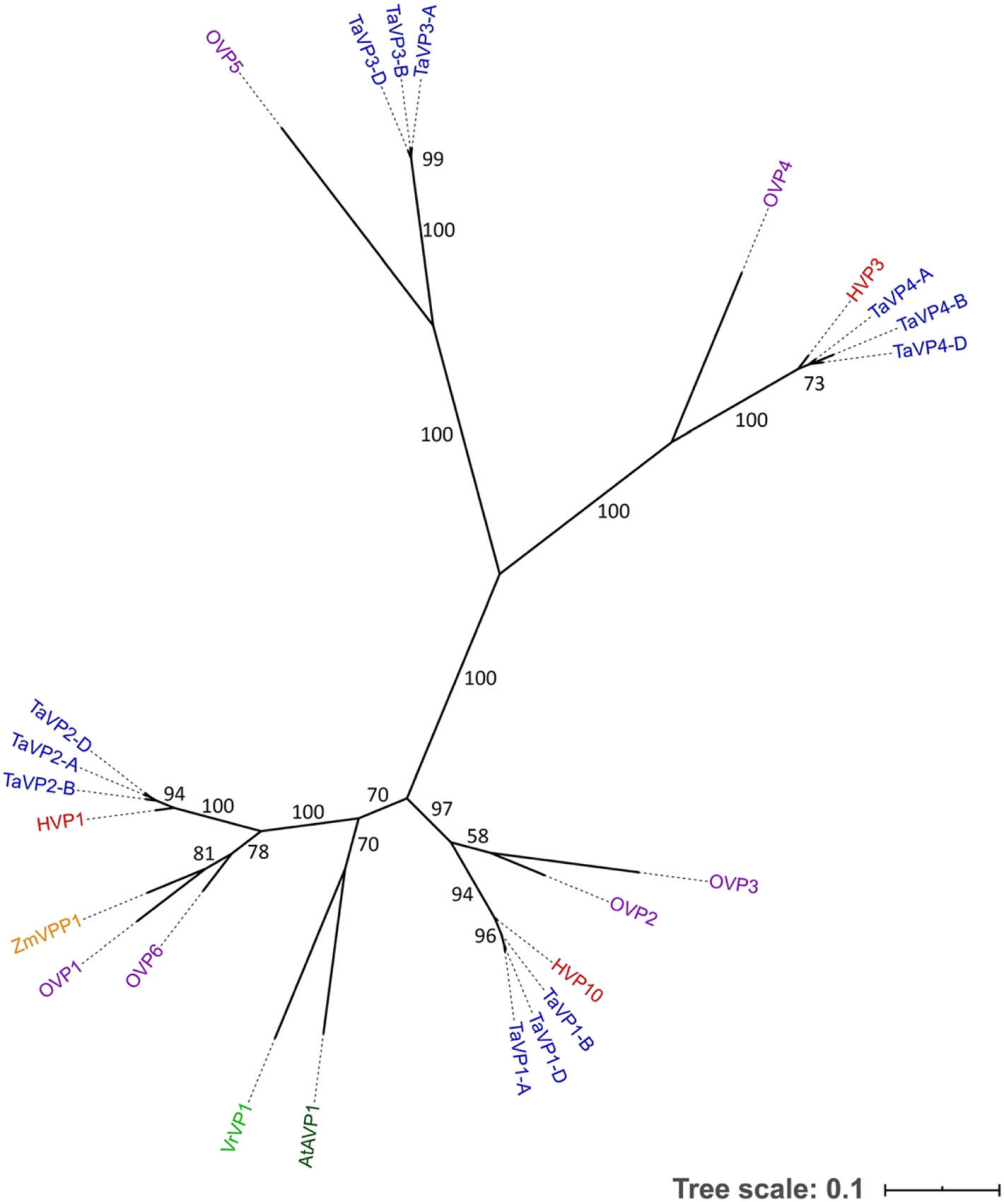
Phylogenetic analysis of type I H^+‐^PPases. Unrooted phylogenetic tree of type I H^+^‐PPase proteins from *Vigna radiata* (VrVP1), *Arabidopsis thaliana* (AVP1), *Hordeum vulgare* (HVP1, HVP10, HVP3), *Zea mays* (ZmVPP1), *Oryza sativa* (OVP1–6), and bread wheat homeologs (TaVP1–TaVP4). Phylogeny was created in MEGA6© via the maximum‐likelihood method and formatted with iTOL. Analysis was validated with 1,000 bootstrap replicates, and bootstrap support for each node is indicated. Scale bar represents 0.1 amino acid substitutions per residue. Dotted lines are for labeling purposes only and are not indicative of branch length

Sequence alignment of TaVP amino acid sequences with the Arabidopsis (AtAVP1, AtAVP2;1, AtVHP2;2), barley (HVP1, HVP10, and HVP3), maize (ZmVPP1 and ZmGPP) and rice (OVP1‐6) H^+^‐PPases (Table S2), as well as the mung bean H^+^‐PPase (VrVP1) for which the protein structure has been comprehensively analyzed (Protein Data Bank accession 4A01), revealed regions containing the PP_i_ binding domain, residues involved in proton translocation, and the transmembrane domains, were highly conserved among all *TaVP* homeologs (Figures 1b S2, and S3). The only noticeable differences were among the TaVP5 proteins, which contained several differences in amino acid sequence compared to the other TaVP proteins, and one mutation at the residue required for proton translocation (E302A) (Figure S3). Proteins of the *TaVP5* homeologs shared high sequence identity with the type II H^+^‐PPases, AVP2 (84%), AVP3 (85%), and ZmGPP (92%) (Figure 1b) and formed a separate clade to the other TaVP homeologs, which grouped with the type I H^+^‐PPases (Figure S4). Within the type I clade, the TaVP1 proteins grouped with the HVP10, OVP2, and OVP4 sequences; the TaVP2 proteins grouped with the HVP1, OVP1, and OVP5 proteins; the TaVP3 proteins grouped with the OVP3 sequence; and the TaVP4 proteins grouped with HVP3 and OVP6 (Figure 2). Arabidopsis AVP1 sits between the TaVP1 and TaVP2 groups.

Further comparison of the wheat *TaVP* homeologs revealed the TaVP5 protein sequences were highly conserved, with only one to two amino acid differences between each TaVP5 homeolog (Figure S3). In comparison, the TaVP1, TaVP2, TaVP3, and TaVP4 protein sequences contained 6, 7, 8, and 11 amino acid differences among homeologs, respectively (Figure S3). RNAseq data from the WheatExp database indicated *TaVP5* homeolog expression to be lower than the other *TaVP*s in the tissue types analyzed (Figure S5). ProteinPredict software also predicted the TaVP5 proteins to localize to the membrane of the Golgi apparatus, while all other TaVPs were predicted to localize to the vacuole membrane (Table 1). Therefore, it is likely that the *TaVP1*, *TaVP2*, *TaVP3*, and *TaVP4* homeologs encode type I H^+^‐PPase proteins, while the *TaVP5* homeologs encode type II H^+^‐PPases.

#### 3.1.1 Synteny analysis

A comparative analysis of the genes adjacent to the TaVPs showed a varying degree of synteny within *T. aestivum* as well as to other grasses and the two selected dicots, Arabidopsis and *Vigna radiate*. Micro‐collinearity of neighboring genes was highly conserved across the homeologous genomes for *TaVP1*, *TaVP2*, and *TaVP5*, whereas around both *TaVP3* and *TaVP4* inversion was observed in the D‐ and B‐genome, respectively (Figures S6–S8). Interestingly, for these two genes, gene orders in barley corresponded more closely to that of the rearranged genomes. Synteny with rice and maize was often observed with the notable exceptions of *TaVP4*; here, none of the adjacent genes were syntenous in rice and TaVP5 where maize did not show any synteny (Figures S6–S8).

### 3.2 Expression of *TaVP* homeologs varies between tissue type, developmental stage, and variety in young wheat seedlings

**FIGURE 3:**
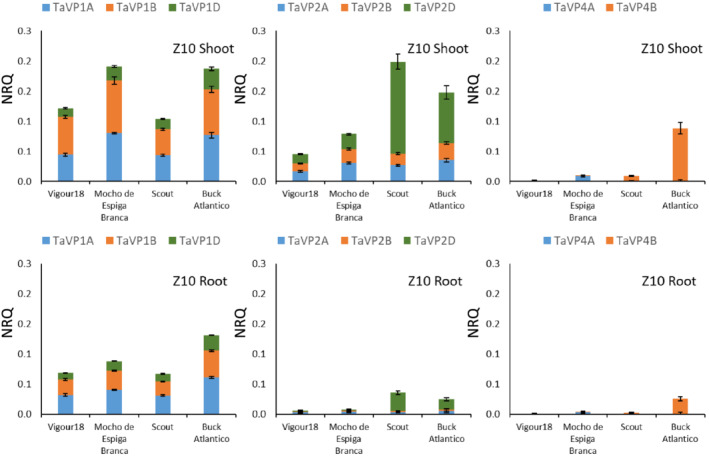
*TaVP* expression profile at seedling stage. Expression of *TaVP* homeologs in shoot (white columns) and roots (gray columns) of 6‐day‐old wheat seedlings (Z10). Expression data displayed as normalized relative quantity (NRQ) for four bread wheat varieties, Vigour18, Mocho de Espiga Branca, Scout, and Buck Atlantico. Values are means of three to five biological replicates and three technical replicates. Error bars represent standard error of the mean

**FIGURE 4:**
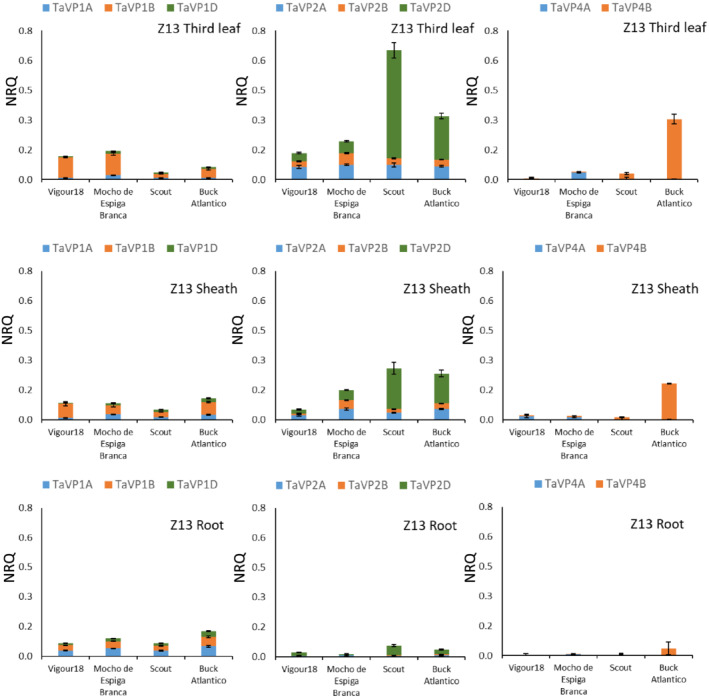
*TaVP* expression profile at third leaf stage. Expression of *TaVP* homeologs in third leaf (white columns), third leaf sheath (dotted columns), and root (gray columns) tissue of plants with fully expanded 3rd leaves (Z13). Expression data displayed as normalized relative quantity (NRQ) for four bread wheat varieties, Vigour18, Mocho de Espiga Branca, Scout, and Buck Atlantico. Values are means of three to five biological replicates and three technical replicates. Error bars represent standard error of the mean

**FIGURE 5:**
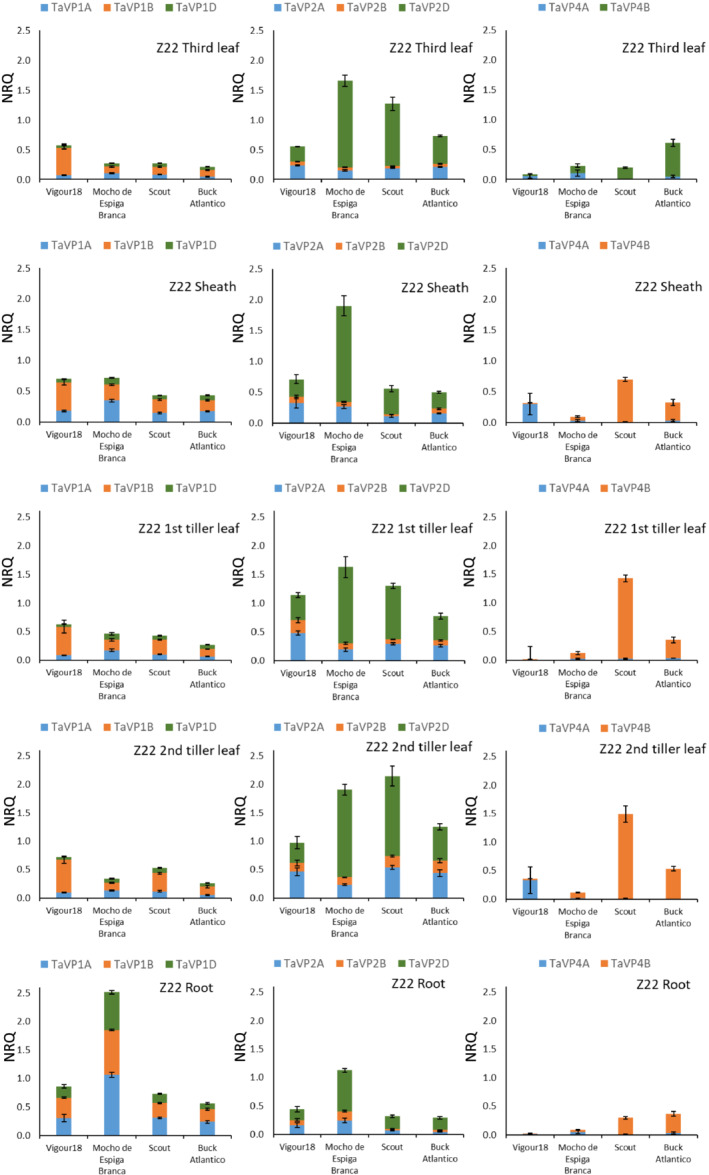
*TaVP* expression profile at second tiller stage. Expression of *TaVP* homeologs in third leaf (white columns), third leaf sheath (dotted columns), first leaf of the first tiller (black columns), first leaf of the second tiller (bricked columns), and root (gray columns) tissue of tillering plants (Z22). Expression data displayed as normalized relative quantity (NRQ) for four bread wheat varieties, Vigour18, Mocho de Espiga Branca, Scout, and Buck Atlantico. Values are means of three to five biological replicates and three technical replicates. Error bars represent standard error of the mean

**FIGURE 6:**
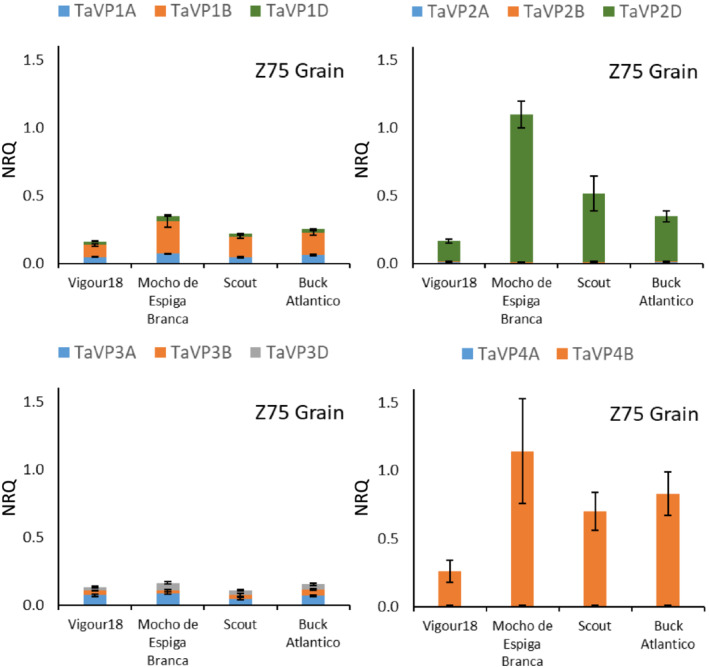
*TaVP* expression profile at grain development stage. Expression of *TaVP* homeologs in the developing grain of field grown plants (Z75). Expression data displayed as normalized relative quantity (NRQ) for four bread wheat varieties, Vigour18, Mocho de Espiga Branca, Scout, and Buck Atlantico. Values are means of three to five biological replicates and three technical replicates. Error bars represent standard error of the mean

In young wheat seedlings at Zadok's stage 10 and 13, expression of *TaVP* homeologs varied significantly between tissues (Figures 3 and 4 and Table S3 and S4). In all tissues at Z10, the *TaVP1* homeologs were expressed twofold higher than the *TaVP2* homeologs, except for *TaVP2‐D*, which was the most highly expressed homeolog in the shoot of Scout (.15 NRQ) and Buck Atlantico (.08 NRQ) (Figure 3; Table S3). At Z10, *TaVP1*, *TaVP2* and *TaVP4* homeologs were, on average, expressed twofold higher in the shoot than the root; however, the overall expression level was low (0–.14 NRQ) compared to the later third leaf (Figure 4; Table S4), tillering (Figure 5; Table S5), or grain development (Figure 6; Table S6) developmental stages. In plants with fully expanded third leaves (Z13), overall *TaVP* expression increased, with *TaVP2‐D* again having the highest level of expression of all *TaVP* homeologs (Figure 4; Table S4). Expression of *TaVP2‐D* varied significantly between varieties at Z13, with high expression in the third leaf of Scout (.55 NRQ) and Buck Atlantico (.22 NRQ) and low expression in Vigour18 (.04 NRQ) and Mocho de Espiga Branca (.01 NRQ) (Figure 4; Table S4). In Vigour18 and Mocho de Espiga Branca, *TaVP1‐B* was expressed threefold higher in the leaf and twofold higher in the sheath than in the root, while no significant differences in *TaVP1‐B* expression between Scout and Buck Atlantico tissues were apparent (Figure 4; Table S4). At both Z10 and Z13, *TaVP4‐B* expression was detected only in Buck Atlantico, while expression of *TaVP4‐A* was only detected in Vigour18 and Mocho de Espiga Branca (Figures 3 and 4; Tables S3 and S4).

### 3.3 Expression of *TaVP* homeologs in tillering plants is dependent on variety

In tillering plants (Z22), *TaVP* expression across all tissues increased approximately eightfold in comparison to Z10 and fivefold compared to Z13 (Figure 5; Table S5). At the second tiller stage, *TaVP1‐A* was expressed threefold to fivefold higher in the root and twofold higher in the sheath, compared to the leaf in all varieties (Figure 5; Table S5). In Mocho de Espiga Branca, expression of *TaVP1‐B* and *TaVP1‐D* was fivefold higher in the root than the leaf tissues, while few significant differences in *TaVP1‐B* and *TaVP1‐D* expression were apparent between tissue types and/or varieties (Figure 5; Table S5). In tillering plants, the majority of *TaVP2* homeologs were expressed greater than twofold higher in leaf tissues than in the root. Exceptions to this were Mocho de Espiga Branca, in which there were no significant differences in *TaVP2‐A* or *TaVP2‐B* expression between tissues, and Vigour18, which had similar levels of *TaVP2‐D* expression (.2–.4 NRQ) across all tissue types (Figure 5; Table S5). Typically, *TaVP2‐D* was the most highly expressed homeolog in all varieties, with expression in all Mocho de Espiga Branca leaf tissue significantly (threefold) higher than all Vigour18 and Buck Atlantico tissues (Figure 5; Table S5). Most varieties had low levels of *TaVP4‐A* and *TaVP4‐B* expression (<.05 NRQ) at the tillering stage, with only Buck Atlantico and Scout having significant levels of *TaVP4‐B* expression (.2–1.6 NRQ) (Figure 5; Table S5).

### 3.4 Expression of *TaVP3* homeologs is restricted to the developing grain

In the developing grain of field grown plants (Z75), all *TaVP* homeologs were expressed at a low level (<.2 NRQ) in all varieties, with the exception of *TaVP2‐D* and *TaVP4‐B* (Figure 6; Table S6). Expression of *TaVP2‐D* was significantly higher in Mocho de Espiga Branca (1.1 NRQ) and Scout (.5 NRQ) than Vigour18 (.15 NRQ), with *TaVP4‐B* expression twofold to fourfold higher in Mocho de Espiga Branca, Scout and Buck Atlantico, compared to Vigour18 (Figure 6; Table S6). Minimal expression (>.01 NRQ) of *TaVP2‐A* and *TaVP2‐B* was detected in the developing grain of all varieties, while no expression of *TaVP4‐A* was detected (Figure 6; Table S6). Expression of *TaVP3* homeologs was not detected in any other tissue types across the four developmental stages analyzed (Figure S9).

### 3.5 *TaVP* homeolog expression is a dynamic trait across tissue types, developmental stages and varieties

**FIGURE 7:**
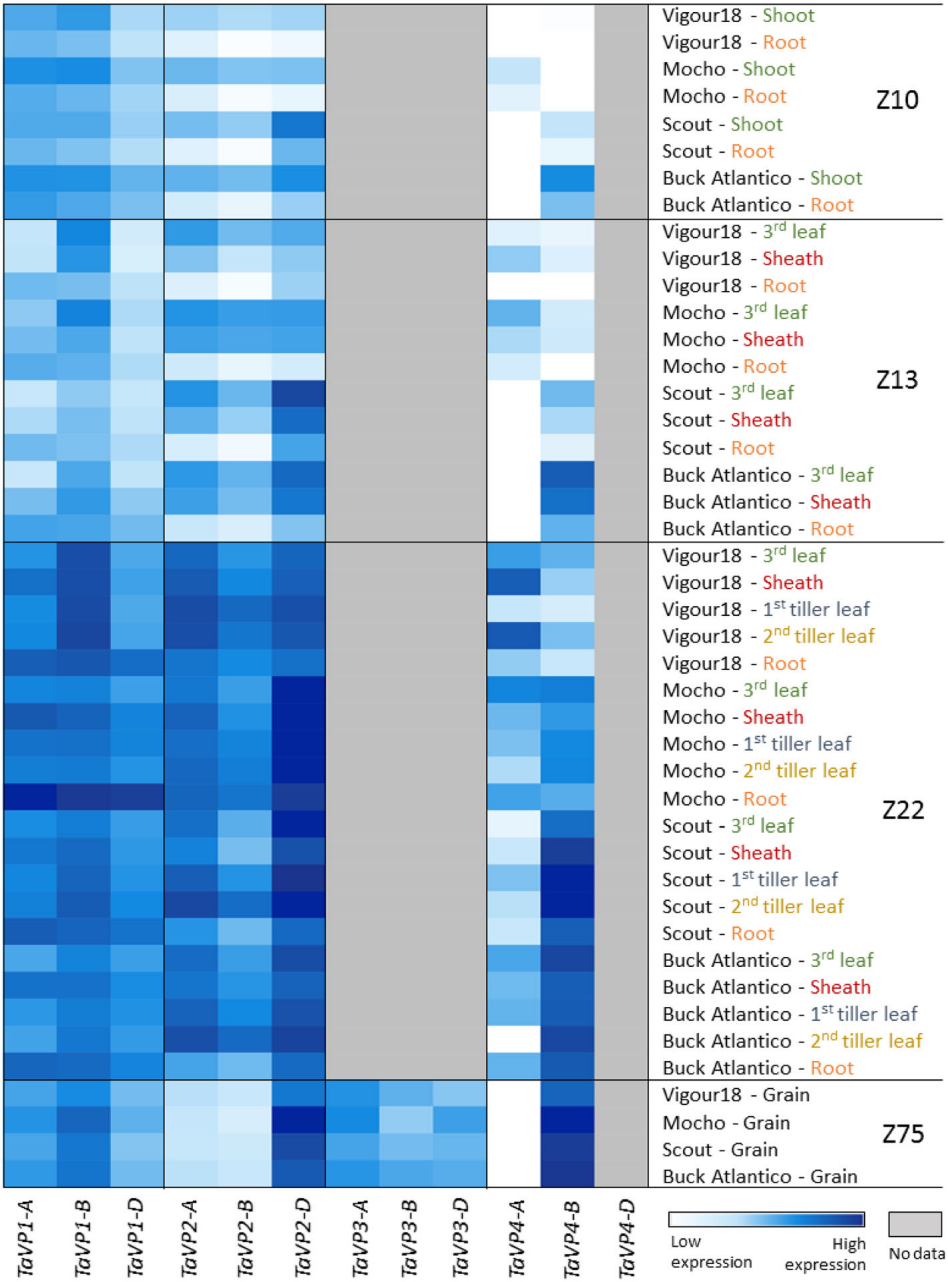
Heat map of *TaVP* gene expression through time. Each column represents an individual *TaVP* homeolog, with developmental stages (Z10, Z13, Z22, and Z75), varieties (Vigour18, Mocho de Espiga Branca [Mocho], Scout, and Buck Atlantico) and tissue types (third leaf, sheath, first tiller leaf, second tiller leaf, root, and grain) displayed along the *y* axis. Colors indicate *TaVP* expression levels, with white and dark blue corresponding to the lowest and highest expression levels, respectively. Gray shading indicates samples for which no qRT‐PCR expression data were obtained

Overall, *TaVP* homeolog expression varied greatly between tissues, developmental stages, and varieties. *TaVP1‐B*, *2‐A*, and *2‐D* were consistently the most highly expressed homeologs across all tissue types and varieties (Figure 7). With the exception of the *TaVP3* homeologs, expression of which were restricted to the developing grain, expression of *TaVP* homeologs generally increased across all tissue types throughout development, from an average of .03 NRQ at Z10 to an average of .28 NRQ at Z22. (Figure 3, 4, 5, 6, 7; Tables S3–S6). The most highly expressed homeologs in the developing grain were *TaVP2‐D* (.2–1.1 NRQ) and *TaVP4‐B* (.3–1.2 NRQ), while expression of all other homeologs was comparatively low (0–.2 NRQ) (Figures 6 and 7). With the exception of Vigour18 leaf tissue, in which *TaVP4‐A* was expressed at a level of .4 NRQ (Figure 5; Table S5), minimal *TaVP4‐A* expression (<.1 NRQ) was detected across all analyzed samples at Z22 (Figures 5 and 7; Table S5). Expression of *TaVP4‐B* was generally specific to Scout and Buck Atlantico, except for the grain development stage (Z75), in which *TaVP4‐B* expression was detected in all four varieties (Figure 3, 4, 5, 6, 7; Table S3–S6).

## 4 DISCUSSION

In this study, we surveyed the bread wheat genome for *H‐PPase* genes. We identified two novel putative bread wheat H^+^‐PPase genes, *TaVP4* and *TaVP5*, along with the three homeologous sequences of both genes from each of the three wheat sub‐genomes (*TaVP4‐A*, *TaVP4‐B*, *TaVP4‐D*, *TaVP5‐A*, *TaVP5‐B*, and *TaVP5‐D*), bringing the total number of identified bread wheat H^+^‐PPase genes to 15. Phylogenetic analysis revealed key groupings between the wheat TaVP homeologs and other cereal H^+^‐PPase proteins.

In the phylogenetic tree, the TaVP1 homeologs clustered with barley HVP10 and the rice OVP2 and OVP4 H^+^‐PPases. Expression of *HVP10* has been shown to be higher in root than shoot tissue (Fukuda et al., 2004; Shavrukov et al., 2013), while *OVP2* and *OVP4* are most highly expressed within the sheath and embryo, respectively (Liu et al., 2010). In this study, it was found that *TaVP1* was typically expressed in vegetative tissues at similar levels in roots and shoots (Figures 3, 4, 5, 6; Tables S3–S6). In contrast, the *TaVP2* genes, which are also predominately expressed in vegetative tissue, are found to have more shoot expression than root (Figures 3, 4, 5, 6; Tables S3–S6). The TaVP2 homeologs grouped with the barley HVP1 and rice OVP1 and OVP5 proteins. Genes encoding the HVP1 (Fukuda et al., 2004) and OVP1 (Liu et al., 2010) proteins have been shown to be predominantly expressed within shoot tissues, while expression of the *OVP5* gene has only been detected at a low level within developing rice grain (Liu et al., 2010). The rice *OVP3* H^+^‐PPase, expression of which is found mainly within seedling roots and embryos (Liu et al., 2010), was the most similar H^+^‐PPase to the *TaVP3* homeologs both in terms of its sequence similarity and expression profile (Figure 6). The *TaVP4* homeologs grouped with the barley *HVP3* whose expression profile has not yet been reported, and the rice *OVP6* protein, which is expressed predominantly within the leaf tissue (Liu et al., 2010). The expression of *TaVP4s* in bread wheat appears to vary across varieties, with some having high expression in all vegetative tissues and others having low expression (Figures 3, 4, 5; Tables S3–S5). While some of the expression patterns within each group are similar across species, overall, there is little correlation between phylogenetic similarities and *TaVP* expression patterns. As gene expression can also be specific to a certain cell type (Byrt et al., 2014), a more comprehensive profiling of tissue and cell types may be required to identify similarities in expression pattern among the *TaVP* homeologs and other cereal H^+^‐PPases.

With 15 *TaVP* genes present in the wheat genome, the variation in gene expression patterns, and differences in protein sequences, it is likely that the TaVP homeologs have different roles in plant development. The *TaVP1* and *TaVP2* homeologs are the most ubiquitous wheat H^+^‐PPase genes and are expressed in all tissue types, varieties, and developmental stages analyzed. While previous research has shown that TaVP1 plays a role in ion accumulation when constitutively expressed in Arabidopsis (Brini et al., 2007), ion accumulation in transgenic *TaVP1* expressing tobacco lines was not enhanced under salinity stress compared to wild‐type, despite showing improved growth (Gouiaa et al., 2012). As such, the function of TaVP1 in ion accumulation remains unclear. Whether the function of TaVP1 when constitutively expressed in Arabidopsis or tobacco is indicative of the native function of this protein in wheat or is the result of being constitutively expressed at a high level in all cells also remains unknown. In several plant species, H^+^‐PPases have been shown to localize to the plasma membrane, in addition to the vacuole membrane, where they are proposed to have a role in transporting sucrose from source to sink tissues (Paez‐Valencia et al., 2011; Pizzio et al., 2015; Regmi et al., 2016). The generally high expression levels of *TaVP1* and *TaVP2* across tissues during the earlier developmental stages indicates they are likely to have important roles in plant development. These roles may include ion sequestration, source to sink transportation of sucrose, or regulation of cytosolic PP_i_ concentrations, which has been shown to have an important role in regulating heterotrophic growth (Ferjani et al., 2011, 2012).

In all analyzed varieties, expression of *TaVP3* homeologs was restricted to the grain during the milk development stage (Z75), consistent with the results of Wang et al. (2009) and transcript data from the WheatEXP database (Pearce et al., 2015). In peach (*Prunus persica*), the *Vp2* H^+^‐PPase has high expression within developing fruit, where it is hypothesized to be involved in the regulation of organic acid and sugar content (Etienne et al., 2002). H^+^‐PPases have also been correlated with Na^+^ accumulation within developing apple (*Malus domestica*) fruit (Yao et al., 2011) and increased ripening and greater phosphorus content in tomato (*Solanum lycopersicum*) fruit (Yang et al., 2014). However, high expression of an H^+^‐PPase gene in rice has been linked to grain chalkiness, an undesirable trait resulting in reduced grain quality (Li et al., 2014). While the expression pattern of *TaVP3* homeologs suggests these genes have developed a specialized role within the grain, further research is required to determine the exact role of these genes in grain development.

Unlike the other *TaVP* genes, expression of *TaVP4* varied significantly between homeologs and varieties. During the early developmental stages, expression of *TaVP4‐B* was lower than the other *TaVP* homeologs, with the exception of Buck Atlantico. Despite considerable efforts, suitable primers for quantifying *TaVP4‐D* expression could not be developed, suggesting this homeolog either has an extremely low level of expression in the tissues and varieties analyzed, or is expressed within a specific cell type within these tissues. For example, the *TaHKT1;5‐D* gene encoding a high‐affinity K^+^ transporter appears to have a low level of expression when analyzed at a tissue level; however, this is due to expression being specific to root stelar cells (Byrt et al., 2014). Cell specific gene expression could account for the generally low levels of *TaVP4* expression detected in the tissues analyzed, and would require more sophisticated techniques, such as laser‐assisted microdissection (Sakai et al., 2018) or fluorescence‐activated cell sorting (Coker et al., 2015) to investigate further. The significant variation in expression between the *TaVP4* homeologs may be due to several reasons, such as homeolog silencing, which can occur as a result of polyploidization (Adams et al., 2003). Homeolog expression can also be coordinately regulated, with expression of a particular homeolog changing in response to increased or reduced expression of another (Lloyd et al., 2017). The large differences in *TaVP4‐B* expression between varieties may also be due to genotypic differences. For example, Buck Atlantico, which had higher expression in Z10 and Z13 compared to other varieties, may contain a different allele for this gene. Further investigation into the expression and allelic diversity of *TaVP4* homeologs in a wider range of wheat varieties is needed to address these questions.

To further investigate the role of the *TaVP* homeologs, changes in *TaVP* expression in response to abiotic stresses, such as salinity, drought, and low nutrient availability, would provide information regarding the potential role of these homeologs in stress tolerance. Analysis of *TaVP* homeolog promoter regions could also provide useful information for identifying wheat varieties with beneficial *TaVP* alleles. For example, the Arabidopsis *AVP1* promoter contains cis‐elements involved in low‐temperature response, sugar response, and pollen specific expression (Pizzio et al., 2015). The rice *OVP3* promoter is known to induce gene expression under cold and oxygen stress (Liu et al., 2010), while genetic variation in the promoter region of the maize *ZmVPP1* gene is correlated with drought tolerance (Wang et al., 2016). In addition, specific amino acid residues which enhance the function of H^+^‐PPases have been identified, such as the E221D mutation, a gain‐of‐function *AVP1* allele in Arabidopsis (Zhen et al., 1997). These known promoter elements and beneficial amino acid residues provide a valuable resource for identifying beneficial *TaVP* alleles for wheat breeding.

In summary, through analyzing newly available wheat reference genomes, six novel *TaVP* gene sequences (*TaVP4‐A*, *TaVP4‐B*, *TaVP4‐D*, *TaVP5‐A*, *TaVP5‐B*, and *TaVP5‐D*) were identified in this study, and full gene sequences were identified for all 15 bread wheat *TaVP* homeologs. Analysis of the type I H^+^‐PPase sequences revealed that *TaVP* homeolog expression is a dynamic trait, which varies between tissue types, developmental stages and wheat varieties. *TaVP3* expression was shown to be specific to the developing grain in all varieties, while *TaVP4* expression varied between varieties and was only present at detectable levels during the later developmental stages. *TaVP1‐B*, *TaVP2‐A*, and *TaVP2‐D* are the most highly expressed *TaVP* homeologs across all tissue types, developmental stages, and varieties, suggesting they have an important role in bread wheat. Further characterization is required to elucidate potential functional differences between TaVP homeologs and to identify beneficial *TaVP* alleles for improving the development, stress tolerance, and yield of bread wheat.

## CONFLICT OF INTEREST

The authors declare that they have no conflict of interest.

## AUTHOR CONTRIBUTIONS

DM identified the homeologs of the *TaVP* genes, as well as performing the qRT‐PCR, and data interpretation; MR performed the synteny analysis and assisted with qRT‐PCR data analysis; UB assisted with the bioinformatics analysis; RS, DP, and SR designed the research and contributed to data analysis and interpretation. All authors contributed to the writing of the manuscript.

## Supporting information


**Table S1:** Details of primer sets used for qRT‐PCR analysis. For each primer set, target gene name, NCBI accession number, forward and reverse primer sequences and amplicon size are provided.
**Table S2:** Gene information of H^+^‐PPase sequences used in phylogenetic analysis. For each gene the species name, gene name, NCBI accession/locus ID (if available), enzyme type and journal reference are provided.
**Table S3:** *TaVP* expression profiles at Zadock's stage Z10. Expression of *TaVP* homeologs was measured in shoots and roots at Zadock's stage Z10 (seedling). Expression data displayed as normalized relative quantity (NRQ) for four bread wheat varieties, Vigour18, Mocho de Espiga Branca, Scout and Buck Atlantico. Values are means of 3–5 biological replicates and 3 technical replicates. Standard error of the mean is presented and different letters indicate statistically significant differences (two‐way ANOVA, P ≤ .05, Tukey's 95% confidence interval) within each gene at that developmental stage.
**Table S4:** *TaVP* expression profiles at Zadock's stage Z13. Expression of *TaVP* homeologs was measured in shoots, roots and third leaf at Zadock's stage Z13 (third leaf unfolded). Expression data displayed as normalized relative quantity (NRQ) for four bread wheat varieties, Vigour18, Mocho de Espiga Branca, Scout and Buck Atlantico. Values are means of 3–5 biological replicates and 3 technical replicates. Standard error of the mean is presented and different letters indicate statistically significant differences (two‐way ANOVA, P ≤ .05, Tukey's 95% confidence interval) within each gene at that developmental stage.
**Table S5:** *TaVP* expression profiles at Zadock's stage Z22. Expression of *TaVP* homeologs was measured in shoots, roots, third leaf, third leaf sheath, first leaf of the first tiller and 1st leaf of the second tiller at Zadock's stage Z22 (tillering – main shoot and 2 tillers). Expression data displayed as normalized relative quantity (NRQ) for four bread wheat varieties, Vigour18, Mocho de Espiga Branca, Scout and Buck Atlantico. Values are means of 3–5 biological replicates and 3 technical replicates. Standard error of the mean is presented and different letters indicate statistically significant differences (two‐way ANOVA, P ≤ .05, Tukey's 95% confidence interval) within each gene at that developmental stage.
**Table S6:** *TaVP* expression profiles at Zadock's stage Z75. Expression of *TaVP* homeologs was measured in developing grain at Zadock's stage Z75 (milk development, medium milk). Expression data displayed as normalized relative quantity (NRQ) for four bread wheat varieties, Vigour18, Mocho de Espiga Branca, Scout and Buck Atlantico. Values are means of 3–5 biological replicates and 3 technical replicates. Standard error of the mean is presented and different letters indicate statistically significant differences (two‐way ANOVA, P ≤ .05, Tukey's 95% confidence interval) within each gene at that developmental stage.
**Figure S1:** Melt curve analysis of *TaVP* homeolog specific primers. Graphs depicting melt curve analysis of amplified products from cDNA (red/blue lines) and no template control (green/yellow) via qRT‐PCR (as previously described). Temperature gradient (60 °C–95 °C) is indicated along the x‐axis, while the y‐axis represents fluorescence from DNA products. Graphs indicate all primers, except *TaVP4‐D*, produce a single product. Peak in *TaVP4‐A* no template control shows primer dimer formation.
**Figure S2:** Complete type I and II H^+^‐PPase amino acid alignment. TaVP homeolog sequences aligned with type I orthologs from *Vigna radiata* (VrVP1), *Arabidopsis thaliana* (AVP1), *Hordeum vulgare* (HVP1, HVP3, HVP10), *Zea mays* (ZmVPP1 [labeled as ZmVP1]) and *Oryza sativa* (OVP1–6), as well as type II orthologs of *A. thaliana* (AVP2, AVP3) and *Z. mays* (ZmGPP [labeled as ZmVP2]). Shading indicates conservation level of amino acid residues where dark blue = 100%, light blue = 80–99%, purple = 60–79% and no color = < 60%.
**Figure S3:** Alignment of 15 identified TaVP homeologs with *V. radiata* H^+^‐PPase (VrVP1) protein sequence. Red shading indicates 100% amino acid conservation. Secondary structure elements are annotated above alignment and are based on the VrVP1 protein structure (Protein Data Bank accession 4A01). Alpha helices and beta pleated sheets are labeled with ‘α’ and ‘β’ respectively. Residues within the VrVP1 sequence known to be involved in proton translocation (stars) and pyrophosphate binding (triangles) are indicated below, as are inner (yellow) and outer (green) transmembrane domains. Alignment was created in Jalview (Waterhouse et al., 2009) using the MUSCLE algorithm (default parameters) and ESPript3.0 (Robert & Gouet, 2014) was used for annotation and visualization of protein features.
**Figure S4:** Phylogenetic analysis of type I and type II H^+^‐PPases. Unrooted phylogenetic tree of type I and II H^+^‐PPase proteins from *V. radiata* (VrVP1), *A. thaliana* (AVP1, AVP2, AVP3), *H. vulgare* (HVP1, HVP10, HVP3), *Z. mays* (ZmVPP1, ZmGPP), *O. sativa* (OVP1–6) and bread wheat homeologs (TaVP1‐TaVP5). Phylogeny was created in MEGA6© via the Maximum‐Likelihood method and formatted with iTOL. Analysis was validated with 1,000 bootstrap replicates and bootstrap support for each node is indicated. Scale bar represents 1.0 amino acid substitutions. Dotted lines are for labeling purposes only and are not included in branch lengths.
**Figure S5:** RNAseq expression data of IWGSCv2 scaffolds. Scaffolds containing (A) *TaVP1* (Traes_7AS_848028906.1, Traes_7BS_55CB27B54.1, Traes_7DS_326DC5875.1), (B) *TaVP2* (Traes_7AL_AA1B5DF85.1, Traes_7BL_FD4254327.4, Traes_7DL_3BA7EF708.2), (C) *TaVP3* (Traes_1AS_90677C42C.1, Traes_1BS_1514DE4E9.2, Traes_1DS_EF07A3CBD.1), and (D) *TaVP5* (Traes_6AL_5F50463BE.2, Traes_6BL_E905C1C95.1, Traes_6DL_FC95036E1.1) genes from the A‐ (blue columns), B‐ (black columns) and D‐genomes (green columns) in grain, leaf, root, spike and stem tissues under control conditions are shown. Data was obtained from the WheatExp database (Pearce et al., 2015) and is displayed as FPKM (Fragments Per Kilobase of transcript per Million mapped reads). Values are means ± standard deviation. As *TaVP4* homeologs are missing from IWGSCv2 assembly, no RNAseq data was available in the WheatExp database.
**Figure S6** Analysis of micro‐collinearity of genes adjacent to TaVP1&2 across grasses and dicots. Protein sequences of the 12 upstream and downstream genes of each TaVP1&2 were obtained and their orthologs were identified by BLASTP in barley, rice, maize, Arabidopsis and mung bean. For each TaVP, orthologues are represented by arrow color; the orientation of the genes is indicated by the direction of the arrow. Genes connected by a solid line retain their proximity, i.e. are microsyntenous, whereas those connected by a dotted line are not.
**Figure S7** Analysis of micro‐collinearity of genes adjacent to TaVP3&4 across grasses and dicots. Protein sequences of the 12 upstream and downstream genes of each TaVP3&4 were obtained and their orthologs were identified by BLASTP in barley, rice, maize, Arabidopsis and mung bean. For each TaVP, orthologues are represented by arrow color; the orientation of the genes is indicated by the direction of the arrow. Genes connected by a solid line retain their proximity, i.e. are microsyntenous, whereas those connected by a dotted line are not.
**Figure S8** Analysis of micro‐collinearity of genes adjacent to TaVP5 across grasses and dicots. Protein sequences of the 12 upstream and downstream genes of each TaVP5 were obtained and their orthologs were identified by BLASTP in barley, rice, maize, Arabidopsis and mung bean. For each TaVP, orthologues are represented by arrow color; the orientation of the genes is indicated by the direction of the arrow. Genes connected by a solid line retain their proximity, i.e. are microsyntenous, whereas those connected by a dotted line are not. For TaVP5‐A the gray box indicates that in Ref1.1 one homeolog, together with its two neighbors, was positioned to an unallocated contig. The position and orientation of these 3 genes on Chr 6A was therefore deduced and remains to be validated.
**Figure S9** Fig: Expression of *TaVP3‐A* in Vigour18 cDNA samples. PCR amplification of *TaVP3‐A* was performed with homeolog specific primers (Supplementary Table 1) and OneTaq polymerase (New England Biosciences, Ipswitch, United States) for 30 cycles (annealing temperature 59 °C). Tissues analyzed include: shoot and root at Z10; 3rd leaf, root and sheath at Z13; 3rd leaf, root, sheath and 1st leaf of the first tiller at Z21; 3rd leaf, root, sheath, 1st leaf of the first tiller and 1st leaf of the second tiller at Z22; developing grain at Z75. M = 100 bp marker (bright bands are 300 and 1,000 bp). Final lane contains water as a negative control (−‘ve). Figure is representative of all *TaVP3* homeologs in Vigour18, as well as the other analyzed varieties.
