# Supplementary material for: Proton‐pumping pyrophosphatase homeolog expression is a dynamic trait in bread wheat ( Triticum aestivum )
Source: Plant Direct. 2021 Oct 7;5(10):e354. doi: 10.1002/pld3.354 (PMC8496507; doi:10.1002/pld3.354)
Supplement: Supplementary file 1 — Table S1: Details of primer sets used for qRT‐PCR analysis. For each primer set, target gene name, NCBI accession number, forward and reverse primer sequences and amplicon size are provided. Table S2: Gene information of H+‐PPase sequences used in phylogenetic analysis. For each gene the species name, gene name, NCBI accession/locus ID (if available), enzyme type and journal reference are provided. Table S3: TaVP expression profiles at Zadock's stage Z10. Expression of TaVP homeologs was measured in shoots and roots at Zadock's stage Z10 (seedling). Expression data displayed as normalized relative quantity (NRQ) for four bread wheat varieties, Vigour18, Mocho de Espiga Branca, Scout and Buck Atlantico. Values are means of 3–5 biological replicates and 3 technical replicates. Standard error of the mean is presented and different letters indicate statistically significant differences (two‐way ANOVA, P ≤ .05, Tukey's 95% confidence interval) within each gene at that developmental stage. Table S4: TaVP expression profiles at Zadock's stage Z13. Expression of TaVP homeologs was measured in shoots, roots and third leaf at Zadock's stage Z13 (third leaf unfolded). Expression data displayed as normalized relative quantity (NRQ) for four bread wheat varieties, Vigour18, Mocho de Espiga Branca, Scout and Buck Atlantico. Values are means of 3–5 biological replicates and 3 technical replicates. Standard error of the mean is presented and different letters indicate statistically significant differences (two‐way ANOVA, P ≤ .05, Tukey's 95% confidence interval) within each gene at that developmental stage. Table S5: TaVP expression profiles at Zadock's stage Z22. Expression of TaVP homeologs was measured in shoots, roots, third leaf, third leaf sheath, first leaf of the first tiller and 1st leaf of the second tiller at Zadock's stage Z22 (tillering – main shoot and 2 tillers). Expression data displayed as normalized relative quantity (NRQ) for four bread wheat varieties, Vigour18, [file PLD3-5-e354-s001.pdf]

Supplementary Material

Supplementary Table 1: Details of primer sets used for qRT-PCR analysis. For each primer set, target gene name, NCBI accession number, forward and reverse primer sequences and amplicon size are provided.

Gene	NCBI accession	Forward primer (5′-3′)	Reverse primer (5′-3′)	Amplicon size (bp)
TaVP1-A	MH376294	GAAGATGACCCAAGGAACCCAG	CAGTCGCAAACAGGGTGGTG	238
TaVP1-B	MH376295	ATAATCTTCCTCTTCCTTGGGTC	GAAAGCGGTGATGAATGCC	243
TaVP1-D	MH376296	GCGGCTCCAAGAACGGCTAC	GCCCTTTGTGCTGAATCCCTC	221
TaVP2-A	MH376297	TCGTTGCTTTCCGCTCTGG	TAAATCCCACCGCCTACACG	193
TaVP2-B	MH376298	TTCATCACCGCTTTCCGCTC	TAAATCCCGCCGCCTACACG	197
TaVP2-D	MH376299	GTTCAGCACCAAGAGTCAGCC	GGAGCGGAAAGCAACGGTAAAC	220
TaVP3-A	MH376300	TCAGCGTCCAACAGCGGCG	GGTCCAGGCAGCAAGCCGAG	296
TaVP3-B	MH376301	ACGCCTACGGTCCCATCAG	CGCTCCGCATCGTCATCG	291
TaVP3-D	MH376302	CGTCCATCTCCTCGTTCGG	CATAGCCGATTACAAGACCTGC	332
TaVP4-A	MH376303	CTCGTCGTCGGCACATTCTTC	GAAGACCAGCGACTCGACGG	293
TaVP4-B	MH376304	CGTCGTCGGCACTTTCTTC	AGACTAGGGACTCCACAGCC	240
TaVP4-D	MH376305	GCTGAGGGAGATGATGCCG	GAAGACCAGCGACTCAACGG	339
TaActin	AY663392	GACAATGGAACCGGAATGGTC	GTGTGATGCCAGATTTTCTCCAT	236
TaCyclophilin	AY456122	CAAGCCGCTGCACTACAAGG	AGGGGACGGTGCAGATGAA	227
TaEFA2	M90077	CAGATTGGCAACGGCTACG	CGGACAGCAAAACGACCAAG	227
TaGAPDH	EU022331	TTCAACATCATTCCAAGCAGCA	CGTAACCCAAAATGCCCTTG	220

Supplementary Table 2: Gene information of H^+-PPase sequences used in phylogenetic analysis. For each gene the species name, gene name, NCBI/locus number (if available), enzyme type and journal reference are provided.

Species	Gene	NCBI/locus number	H^+-PPase type	Reference
Arabidopsis thaliana	*AVP1*	BAA32210/*AT1G15690.1*	I	Sarafian et al. (1992)
Arabidopsis thaliana	*AtAVP2;1*	AAF31163.1/*AT1G78920.1*	II	Drozdowicz et al. (2000)
Arabidopsis thaliana	*AtAVP2;2*	AAG09080.1/*AT1G16780.2*	II	Shimaoka et al. (2004)
Hordeum vulgare	*HVP1*	BAB18681.1/ HORVU7Hr1G115540.5	I	Fukuda et al. (2004)
Hordeum vulgare	*HVP10*	BAA02717.2/ *HORVU7Hr1G028910.3*	I	Tanaka et al. (1993)
Hordeum vulgare	*HVP3*	BAJ93792.1/ *HORVU1Hr1G061140*	I	Shavrukov Y (2014)
Oryza sativa	*OVP1*	BAA08232.1/ *Os06t0644200-01*	I	Sakakibara et al. (1996)
Oryza sativa	*OVP2*	BAA08233.1/ *Os06t0178900-01*	I	Sakakibara et al. (1996)
Oryza sativa	*OVP3*	BAD02276.1/ *Os02t0802500-01*	I	Choura and Rebai (2005)
Oryza sativa	*OVP4*	AAQ19328.1/ *Os01t0337500-00*	I	Choura and Rebai (2005)
Oryza sativa	*OVP5*	BAG98020.1/ *Os05t0156900-01*	I	Choura and Rebai (2005)
Oryza sativa	*OVP6*	BAS81426.1/ *LOC_Os02g09150.1*	I	Muto et al. (2014)
Triticum aestivum	*TaVP1-A*	MH376294/ *TraesCS7A02G141300.2*	I	Current study
Triticum aestivum	*TaVP1-B*	MH376295/ *TraesCS7B02G042600.1*	I	Current study
Triticum aestivum	*TaVP1-D*	MH376296/ *TraesCS7D02G142600.2*	I	Current study
Triticum aestivum	*TaVP2-A*	MH376297/ *TraesCS7A02G517700.2*	I	Current study
Triticum aestivum	*TaVP2-B*	MH376298/ *TraesCS7B02G433800.1*	I	Current study
Triticum aestivum	*TaVP2-D*	MH376299/ *TraesCS7D02G507600.1*	I	Current study
Triticum aestivum	*TaVP3-A*	MH376300/ *TraesCS1A02G107200.1*	I	Current study
Triticum aestivum	*TaVP3-B*	MH376301/ *TraesCS1B02G124600.1*	I	Current study
Triticum aestivum	*TaVP3-D*	MH376302/ *TraesCS1D02G107600.1*	I	Current study
Triticum aestivum	*TaVP4-A*	MH376303/ *TraesCS1A02G253600.1*	I	Current study

Triticum aestivum	*TaVP4-B*	MH376304/ *TraesCS1B02G264600.1*	I	Current study
Triticum aestivum	*TaVP4-D*	MH376305/ *TraesCS1D02G253200.1*	I	Current study
Triticum aestivum	*TaVP5-A*	MH376306/ *TraesCSU02G080600.1*	II	Current study
Triticum aestivum	*TaVP5-B*	MH376307/ *TraesCS6B02G376400.1*	II	Current study
Triticum aestivum	*TaVP5-D*	MH376308/ *TraesCS6D02G326300.1*	II	Current study
Vigna radiata	*VrVP2*	BAA23649/ *Vradi07g20720*	I	Nakanishi & Maeshima (1998)
Zea mays	*ZmVPP1*	CAG29369.1/ *Zm00001d015569_P001*	I	Wisniewski & Rogowsky (2004)
Zea mays	*ZmGPP*	ABK51382.1/ *Zm00001d030996_P002*	II	Yue et al. (2008)

Supplementary Table 3: *TaVP* expression profiles at Zadock's stage Z10. Expression of *TaVP* homeologs was measured in shoots and roots at Zadock's stage Z10 (seedling). Expression data displayed as normalised relative quantity (NRQ) for four bread wheat varieties, Vigour18, Mocho de Espiga Branca, Scout and Buck Atlantico. Values are means of 3-5 biological replicates and 3 technical replicates. Standard error of the mean is presented and different letters indicate statistically significant differences (two-way ANOVA, P ≤ 0.05, Tukey's 95 % confidence interval) within each gene at that developmental stage.

**Z10 Seedling** *TaVP1* homeolog expression profile (NRQ)				
**Accession**	**Tissue**	***TaVP1A***	***TaVP1B***	***TaVP1D***
Vigour18	Shoot	0.044 ± 0.002^{b}	0.063 ± 0.022^{c}	0.014 ± 0.001^{ab}
	Root	0.032 ± 0.002^{a}	0.026 ± 0.002^{a}	0.011 ± 0.001^{a}
Mocho de Espiga Branca	Shoot	0.080 ± 0.001^{d}	0.087 ± 0.006^{d}	0.024 ± 0.001^{c}
	Root	0.040 ± 0.001^{ab}	0.032 ± 0.001^{ab}	0.016 ± 0.001^{ab}
Scout	Shoot	0.043 ± 0.002^{b}	0.043 ± 0.002^{b}	0.018 ± 0.001^{b}
	Root	0.031 ± 0.001^{a}	0.023 ± 0.001^{a}	0.013 ± 0.001^{ab}
Buck Atlantico	Shoot	0.077 ± 0.005^{d}	0.076 ± 0.005^{d}	0.034 ± 0.003^{d}
	Root	0.061 ± 0.002^{c}	0.044 ±0.001^{b}	0.026 ± 0.001^{a}

**Z10 Seedling** *TaVP2* homeolog expression profile (NRQ)				
**Accession**	**Tissue**	***TaVP2A***	***TaVP2B***	***TaVP2D***
Vigour18	Shoot	0.016 ± 0.002^{b}	0.013 ± 0.001^{b}	0.016 ± 0.001^{ab}
	Root	0.003 ± 0.000^{a}	0.001 ± 0.000^{a}	0.002 ± 0.000^{a}
Mocho de Espiga Branca	Shoot	0.031 ± 0.002^{cd}	0.023 ± 0.002^{cd}	0.025 ± 0.001^{ab}
	Root	0.004 ± 0.001^{a}	0.001 ± 0.000^{a}	0.002 ± 0.000^{ab}
Scout	Shoot	0.027 ± 0.001^{c}	0.019 ± 0.002^{c}	0.153 ± 0.012^{d}
	Root	0.003 ± 0.000^{a}	0.001 ± 0.000^{a}	0.031 ± 0.003^{b}
Buck Atlantico	Shoot	0.036 ± 0.003^{d}	0.028 ± 0.002^{d}	0.084 ± 0.011^{c}
	Root	0.005 ± 0.000^{a}	0.002 ± 0.000^{a}	0.018 ± 0.003^{ab}

**Z10 Seedling**<br>*TaVP4* homeolog expression profile (NRQ)			
**Accession**	**Tissue**	***TaVP4A***	***TaVP4B***
Vigour18	Shoot	0.000 ± 0.000	0.001 ± 0.000[a]
	Root	0.000 ± 0.000	0.001 ± 0.000[a]
Mocho de Espiga Branca	Shoot	0.009 ± 0.002[b]	0.000 ± 0.000[a]
	Root	0.003 ± 0.000[a]	0.000 ± 0.000[a]
Scout	Shoot	0.000 ± 0.000	0.009 ± 0.001[ab]
	Root	0.000 ± 0.000	0.002 ± 0.000[a]
Buck Atlantico	Shoot	0.000 ± 0.000	0.088 ± 0.010[c]
	Root	0.000 ± 0.000	0.026 ± 0.003[b]

Supplementary Table 4: *TaVP* expression profiles at Zadock's stage Z13. Expression of *TaVP* homeologs was measured in shoots, roots and third leaf at Zadock's stage Z13 (third leaf unfolded). Expression data displayed as normalised relative quantity (NRQ) for four bread wheat varieties, Vigour18, Mocho de Espiga Branca, Scout and Buck Atlantico. Values are means of 3-5 biological replicates and 3 technical replicates. Standard error of the mean is presented and different letters indicate statistically significant differences (two-way ANOVA, P ≤ 0.05, Tukey's 95 % confidence interval) within each gene at that developmental stage.

**Z13 Main shoot and two tillers**<br>*TaVP1* homeolog expression profile (NRQ)				
**Accession**	**Tissue**	***TaVP1A***	***TaVP1B***	***TaVP1D***
Vigour18	3rd leaf	0.009 ± 0.001^{a}	0.103 ± 0.006^{d}	0.005 ± 0.000^{a}
	Sheath	0.010 ± 0.001^{a}	0.071 ± 0.011^{c}	0.004 ± 0.001^{a}
	Root	0.029 ± 0.001^{c}	0.027 ± 0.001^{a}	0.011 ± 0.001^{ab}
Mocho de Espiga Branca	3rd leaf	0.020 ± 0.001	0.109 ± 0.009^{d}	0.013 ± 0.001^{bc}
	Sheath	0.028 ± 0.002^{c}	0.045 ± 0.007^{abc}	0.011 ± 0.001^{ab}
	Root	0.040 ± 0.002^{d}	0.036 ± 0.001^{ab}	0.013 ± 0.002^{bc}
Scout	3rd leaf	0.008 ± 0.000^{a}	0.020 ± 0.002^{a}	0.008 ± 0.000^{ab}
	Sheath	0.014 ± 0.001^{ab}	0.026 ± 0.002^{a}	0.011 ± 0.001^{ab}
	Root	0.029 ± 0.002^{c}	0.025 ± 0.003^{a}	0.014 ± 0.001^{bc}
Buck Atlantico	3rd leaf	0.008 ± 0.001^{a}	0.045 ± 0.002^{abc}	0.010 ± 0.001^{ab}
	Sheath	0.027 ± 0.003^{c}	0.062 ± 0.004^{bc}	0.020 ± 0.002^{cd}
	Root	0.051 ± 0.004^{d}	0.048 ± 0.005^{abc}	0.028 ± 0.004^{d}

**Z13 Main shoot and two tillers**<br>*TaVP2* homeolog expression profile (NRQ)				
**Accession**	**Tissue**	***TaVP2A***	***TaVP2B***	***TaVP2D***
Vigour18	3rd leaf	0.063 ± 0.009^{d}	0.028 ± 0.003^{cd}	0.043 ± 0.005^{a}
	Sheath	0.023 ± 0.004^{cd}	0.009 ± 0.002^{ab}	0.020 ± 0.002^{a}
	Root	0.004 ± 0.001^{a}	0.001 ± 0.000^{a}	0.017 ± 0.000^{a}
Mocho de Espiga Branca	3rd leaf	0.075 ± 0.004^{d}	0.059 ± 0.003^{f}	0.060 ± 0.004^{a}
	Sheath	0.054 ± 0.005^{cd}	0.046 ± 0.003^{ef}	0.051 ± 0.002^{a}
	Root	0.006 ± 0.001^{a}	0.002 ± 0.001^{a}	0.005 ± 0.001^{a}
Scout	3rd leaf	0.074 ± 0.010^{d}	0.032 ± 0.002^{d}	0.545 ± 0.039^{d}
	Sheath	0.036 ± 0.002^{bc}	0.018 ± 0.002^{bc}	0.206 ± 0.029^{bc}
	Root	0.005 ± 0.000^{a}	0.002 ± 0.000^{a}	0.050 ± 0.005^{a}
Buck Atlantico	3rd leaf	0.066 ± 0.004^{d}	0.034 ± 0.001^{de}	0.220 ± 0.015^{c}
	Sheath	0.054 ± 0.003^{cd}	0.028 ± 0.002^{cd}	0.152 ± 0.018^{b}
	Root	0.008 ± 0.001^{a}	0.004 ± 0.000^{a}	0.023 ± 0.002^{a}

**Z13 Main shoot and two tillers**<br>*TaVP4* homeolog expression profile (NRQ)			
**Accession**	**Tissue**	***TaVP4A***	***TaVP4B***
Vigour18	3rd leaf	0.003 ± 0.000^{a}	0.002 ± 0.000^{a}
	Sheath	0.019 ± 0.002^{ab}	0.004 ± 0.000^{a}
	Root	0.001 ± 0.000^{a}	0.001 ± 0.000^{a}
Mocho de Espiga Branca	3rd leaf	0.034 ± 0.001^{c}	0.005 ± 0.000^{a}
	Sheath	0.014 ± 0.001^{b}	0.006 ± 0.000^{a}
	Root	0.005 ± 0.000^{a}	0.000 ± 0.000^{a}
Scout	3rd leaf	0.000 ± 0.000	0.029 ± 0.005^{a}
	Sheath	0.000 ± 0.000	0.014 ± 0.004^{a}
	Root	0.000 ± 0.000	0.003 ± 0.001^{a}
Buck Atlantico	3rd leaf	0.000 ± 0.000	0.304 ± 0.025^{c}
	Sheath	0.000 ± 0.000	0.183 ± 0.033^{b}

	Root	0.000 ± 0.000	0.036 ± 0.002[a]

Supplementary Table 5: *TaVP* expression profiles at Zadock's stage Z22. Expression of *TaVP* homeologs was measured in shoots, roots, third leaf, third leaf sheath, first leaf of the first tiller and 1st leaf of the second tiller at Zadock's stage Z22 (tillering – main shoot and 2 tillers). Expression data displayed as normalised relative quantity (NRQ) for four bread wheat varieties, Vigour18, Mocho de Espiga Branca, Scout and Buck Atlantico. Values are means of 3-5 biological replicates and 3 technical replicates. Standard error of the mean is presented and different letters indicate statistically significant differences (two-way ANOVA, P ≤ 0.05, Tukey's 95 % confidence interval) within each gene at that developmental stage.

**Z22 Main shoot and two tillers** *TaVP1* homeolog expression profile (NRQ)				
**Accession**	**Tissue**	***TaVP1A***	***TaVP1B***	***TaVP1D***
Vigour18	3rd leaf	0.074 ± 0.0092^{ab}	0.455 ± 0.070^{fg}	0.045 ± 0.006^{a}
	Sheath	0.182 ± 0.0132^{abcde}	0.464 ± 0.048^{fg}	0.053 ± 0.006^{a}
	1st tiller leaf	0.088 ± 0.0042^{ab}	0.499 ± 0.110^{efg}	0.044 ± 0.004^{a}
	2nd tiller leaf	0.097 ± 0.0075^{abcd}	0.576 ± 0.067^{g}	0.049 ± 0.003^{a}
	Root	0.301 ± 0.0631^{bde}	0.360 ± 0.012^{defg}	0.198 ± 0.031^{a}
Mocho de Espiga Branca	3rd leaf	0.105 ± 0.0090^{abcd}	0.114 ± 0.006^{ab}	0.055 ± 0.007^{a}
	Sheath	0.348 ± 0.0199^{e}	0.258 ± 0.012^{bcde}	0.110 ± 0.008^{a}
	1st tiller leaf	0.173 ± 0.0224^{abcde}	0.184 ± 0.021^{abac}	0.107 ± 0.022^{a}
	2nd tiller leaf	0.128 ± 0.0097^{abcd}	0.138 ± 0.011^{ab}	0.074 ± 0.008^{a}
	Root	1.065 ± 0.0455^{f}	0.787 ± 0.011^{h}	0.663 ± 0.031^{b}
Scout	3rd leaf	0.085 ± 0.0056^{abc}	0.129 ± 0.011^{ab}	0.058 ± 0.003^{a}
	Sheath	0.147 ± 0.0137^{abcd}	0.218 ± 0.012^{abcde}	0.065 ± 0.004^{a}
	1st tiller leaf	0.103 ± 0.0064^{abcd}	0.254 ± 0.009^{abcd}	0.074 ± 0.005^{a}
	2nd tiller leaf	0.118 ± 0.0140^{abcd}	0.318 ± 0.015^{cdef}	0.091 ± 0.011^{a}
	Root	0.312 ± 0.0121^{e}	0.251 ± 0.009^{abcde}	0.168 ± 0.012^{a}
Buck Atlantico	3rd leaf	0.047 ± 0.0084^{a}	0.110 ± 0.007^{a}	0.056 ± 0.005^{a}
	Sheath	0.174 ± 0.0073^{abcde}	0.177 ± 0.011^{abc}	0.085 ± 0.008^{a}
	1st tiller leaf	0.067 ± 0.0040^{a}	0.130 ± 0.004^{ab}	0.077 ± 0.006^{a}
	2nd tiller leaf	0.052 ± 0.0076^{a}	0.147 ± 0.019^{ab}	0.062 ± 0.004^{a}
	Root	0.241 ± 0.0231^{bcde}	0.215 ± 0.017^{abcd}	0.106 ± 0.009^{a}

Z22 Main shoot and two tillers<br>*TaVP2* homeolog expression profile (NRQ)				
**Accession**	**Tissue**	***TaVP2A***	***TaVP2B***	***TaVP2D***
Vigour18	3rd leaf	0.237 ± 0.013^{abcdef}	0.070 ± 0.003^{ab}	0.250 ± 0.002^{ab}
	Sheath	0.327 ± 0.088^{abcdef}	0.096 ± 0.027^{ab}	0.290 ± 0.070^{ab}
	1st tiller leaf	0.484 ± 0.034^{efg}	0.223 ± 0.047^{bcd}	0.436 ± 0.041^{abc}
	2nd tiller leaf	0.462 ± 0.071^{defg}	0.155 ± 0.051^{abcd}	0.356 ± 0.108^{ab}
	Root	0.167 ± 0.038^{abcd}	0.091 ± 0.022^{ab}	0.184 ± 0.044^{a}
Mocho de Espiga Branca	3rd leaf	0.147 ± 0.018^{abcd}	0.057 ± 0.006^{a}	1.457 ± 0.095^{e}
	Sheath	0.264 ± 0.034^{bcdef}	0.078 ± 0.007^{ab}	1.562 ± 0.162^{e}
	1st tiller leaf	0.196 ± 0.032^{abcd}	0.108 ± 0.021^{abc}	1.324 ± 0.184^{de}
	2nd tiller leaf	0.235 ± 0.017^{abcdef}	0.128 ± 0.005^{abcd}	1.544 ± 0.097^{e}
	Root	0.249 ± 0.037^{abcdef}	0.161 ± 0.016^{bcd}	0.717 ± 0.030^{abcd}
Scout	3rd leaf	0.187 ± 0.012^{abcd}	0.038 ± 0.003^{a}	1.045 ± 0.112^{cde}
	Sheath	0.112 ± 0.023^{abc}	0.026 ± 0.003^{a}	0.420 ± 0.047^{abc}
	1st tiller leaf	0.298 ± 0.016^{cdef}	0.075 ± 0.006^{ab}	0.927 ± 0.044^{bcde}
	2nd tiller leaf	0.534 ± 0.036^{g}	0.202 ± 0.019^{cd}	1.410 ± 0.176^{e}
	Root	0.072 ± 0.012^{ab}	0.030 ± 0.005^{a}	0.220 ± 0.023^{a}
Buck Atlantico	3rd leaf	0.206 ± 0.009^{abcde}	0.057 ± 0.006^{a}	0.466 ± 0.012^{abc}
	Sheath	0.160 ± 0.008^{abcd}	0.071 ± 0.011^{ab}	0.269 ± 0.015^{ab}
	1st tiller leaf	0.265 ± 0.024^{bcdef}	0.092 ± 0.015^{ab}	0.416 ± 0.054^{abc}
	2nd tiller leaf	0.435 ± 0.057^{fg}	0.218 ± 0.037^{d}	0.600 ± 0.054^{abc}
	Root	0.049 ± 0.006^{a}	0.029 ± 0.001^{a}	0.218 ± 0.022^{a}

Z22 Main shoot and two tillers<br>*TaVP4* homeolog expression profile (NRQ)			
**Accession**	**Tissue**	***TaVP4A***	***TaVP4B***
Vigour18	3rd leaf	0.057 ± 0.0400[a]	0.036 ± 0.000[a]
	Sheath	0.302 ± 0.1743[a]	0.018 ± 0.001[a]
	1st tiller leaf	0.008 ± 0.0048[a]	0.005 ± 0.000[a]
	2nd tiller leaf	0.330 ± 0.2333[a]	0.026 ± 0.000[a]
	Root	0.019 ± 0.0108[a]	0.008 ± 0.003[a]
Mocho de Espiga Branca	3rd leaf	0.106 ± 0.0521[a]	0.122 ± 0.030[a]
	Sheath	0.031 ± 0.0160[a]	0.064 ± 0.011[a]
	1st tiller leaf	0.025 ± 0.0145[a]	0.095 ± 0.025[a]
	2nd tiller leaf	0.013 ± 0.0074[a]	0.102 ± 0.011[a]
	Root	0.052 ± 0.0266[a]	0.040 ± 0.008[a]
Scout	3rd leaf	0.003 ± 0.0014[a]	0.197 ± 0.015[ab]
	Sheath	0.008 ± 0.0043[a]	0.690 ± 0.036[b]
	1st tiller leaf	0.024 ± 0.0135[a]	1.402 ± 0.059[c]
	2nd tiller leaf	0.012 ± 0.0055[a]	1.482 ± 0.147[c]
	Root	0.008 ± 0.0040[a]	0.294 ± 0.024[ab]
Buck Atlantico	3rd leaf	0.047 ± 0.0217[a]	0.570 ± 0.059[b]
	Sheath	0.029 ± 0.0172[a]	0.296 ± 0.051[ab]
	1st tiller leaf	0.033 ± 0.0118[a]	0.319 ± 0.048[ab]
	2nd tiller leaf	0.000 ± 0.0000[a]	0.533 ± 0.042[b]
	Root	0.034 ± 0.0175[a]	0.336 ± 0.044[ab]

Supplementary Table 6: *TaVP* expression profiles at Zadock's stage Z75. Expression of *TaVP* homeologs was measured in developing grain at Zadock's stage Z75 (milk development, medium milk). Expression data displayed as normalised relative quantity (NRQ) for four bread wheat varieties, Vigour18, Mocho de Espiga Branca, Scout and Buck Atlantico. Values are means of 3-5 biological replicates and 3 technical replicates. Standard error of the mean is presented and different letters indicate statistically significant differences (two-way ANOVA, P ≤ 0.05, Tukey's 95 % confidence interval) within each gene at that developmental stage.

Z75 Grain filling
TaVP1 homeolog expression profile (NRQ)

Accession	Tissue	*TaVP1A*	*TaVP1B*	*TaVP1D*
Vigour18	Grain	0.049 ± 0.004[a]	0.088 ± 0.009[a]	0.028 ± 0.001[ab]
Mocho de Espiga Branca	Grain	0.074 ± 0.004[b]	0.241 ± 0.046[b]	0.036 ± 0.004[b]
Scout	Grain	0.048 ± 0.004[a]	0.150 ± 0.013[ab]	0.023 ± 0.001[a]
Buck Atlantico	Grain	0.064 ± 0.006[ab]	0.161 ± 0.017[ab]	0.028 ± 0.003[ab]

Z75 Grain filling
TaVP2 homeolog expression profile (NRQ)

Accession	Tissue	*TaVP2A*	*TaVP2B*	*TaVP2D*
Vigour18	Grain	0.012 ± 0.001[a]	0.008 ± 0.001[a]	0.148 ± 0.014[a]
Mocho de Espiga Branca	Grain	0.009 ± 0.001[a]	0.005 ± 0.000[a]	1.084 ± 0.099[c]
Scout	Grain	0.008 ± 0.001[a]	0.007 ± 0.000[a]	0.504 ± 0.130[b]
Buck Atlantico	Grain	0.011 ± 0.001[a]	0.008 ± 0.001[a]	0.330 ± 0.043[ab]

Z75 Grain filling
TaVP3 homeolog expression profile (NRQ)

Accession	Tissue	*TaVP3A*	*TaVP3B*	*TaVP3D*
Vigour18	Grain	0.075 ± 0.009[ab]	0.036 ±0.002[a]	0.022 ± 0.004[a]
Mocho de Espiga Branca	Grain	0.090 ± 0.010[b]	0.019 ± 0.008[a]	0.055 ± 0.009[b]
Scout	Grain	0.049 ± 0.010[a]	0.028 ± 0.010[a]	0.032 ± 0.005[ab]

Buck Atlantico	Grain	0.071 ± 0.007[ab]	0.045 ± 0.007[a]	0.040 ± 0.010[ab]

**Z75 Grain filling** *TaVP1* homeolog expression profile (NRQ)			
**Accession**	**Tissue**	***TaVP4A***	***TaVP4B***
Vigour18	Grain	0.000 ± 0.000	0.081 ± 0.000[a]
Mocho de Espiga Branca	Grain	0.000 ± 0.000	0.388 ± 0.000[b]
Scout	Grain	0.000 ± 0.000	0.140 ± 0.000[ab]
Buck Atlantico	Grain	0.000 ± 0.000	0.159 ± 0.000[ab]

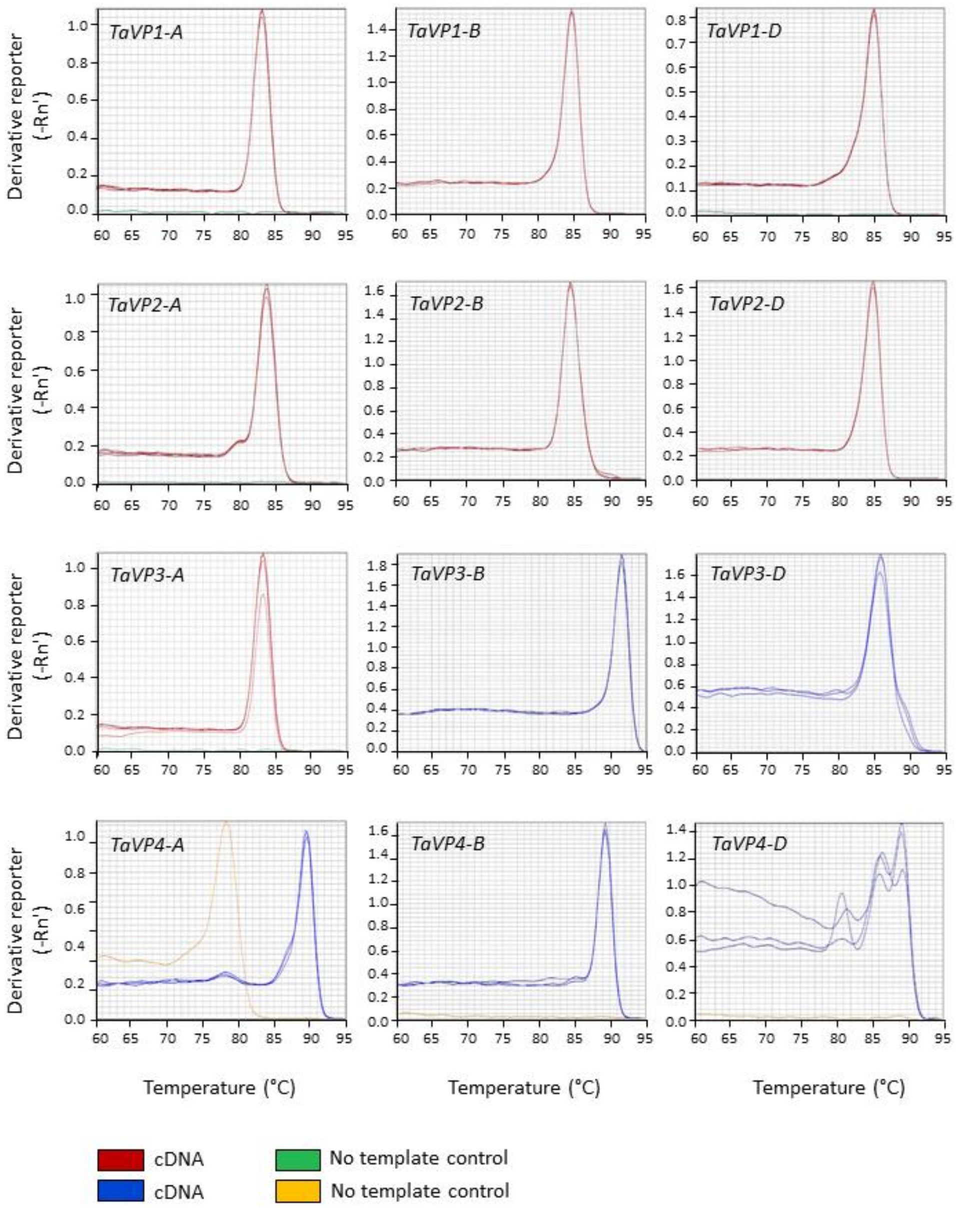

Supplementary Figure 1: Melt curve analysis of *TaVP* homeolog specific primers. Graphs depicting melt curve analysis of amplified products from cDNA (red/blue lines) and no template control (green/yellow) via qRT-PCR (as previously described). Temperature gradient (60°C-95°C) is indicated along the x-axis, while the y-axis represents fluorescence from DNA products. Graphs indicate all primers, except *TaVP4-D*, produce a single product. Peak in *TaVP4-A* no template control shows primer dimer formation

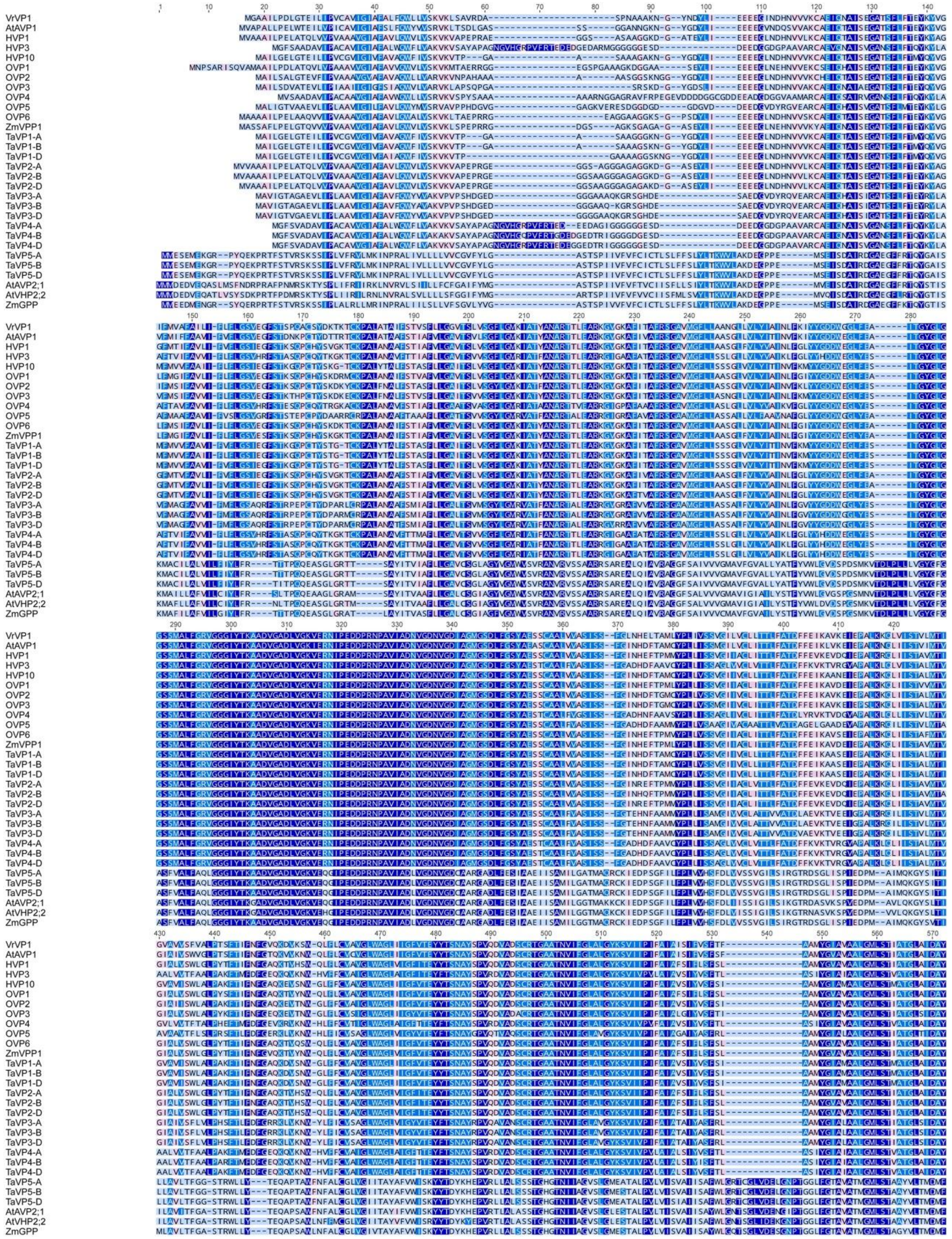
VrVP1
AtAVP1
HVP1
HVP3
HVP10
OVP1
OVP2
OVP3
OVP4
OVP5
OVP6
ZmVPP1
TaVP1-A
TaVP1-B
TaVP1-D
TaVP2-A
TaVP2-B
TaVP2-D
TaVP3-A
TaVP3-B
TaVP3-D
TaVP4-A
TaVP4-B
TaVP4-D
TaVP5-A
TaVP5-B
TaVP5-D
AtAVP2;1
AtVHP2;2
ZmGPP

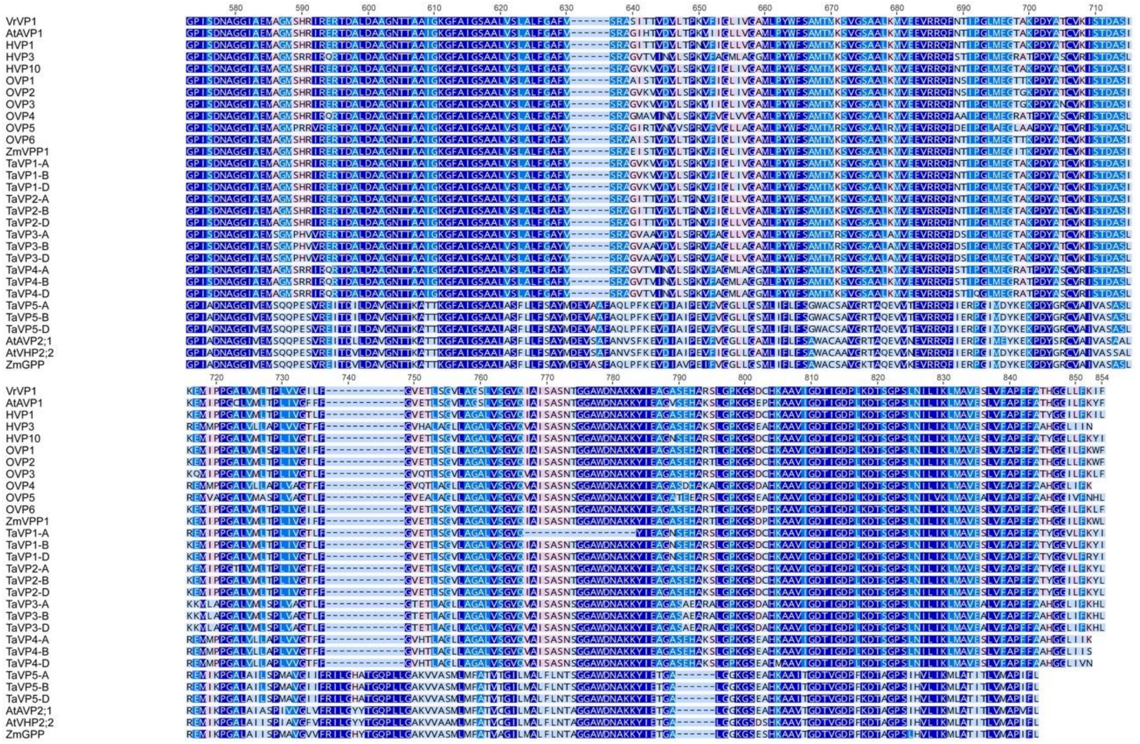

Supplementary Figure 2: Complete type I and II H^+-PPase amino acid alignment. TaVP homeolog sequences aligned with type I orthologs from *Vigna radiata* (VrVP1), *Arabidopsis thaliana* (AVP1), *Hordeum vulgare* (HVP1, HVP3, HVP10), *Zea mays* (ZmVPP1) and *Oryza sativa* (OVP1-6), as well as type II orthologs of *Arabidopsis thaliana* (AVP2, AVP3) and *Zea mays* (ZmGPP). Shading indicates conservation level of amino acid residues where dark blue = 100 %, light blue = 80-99 %, purple = 60-79% and no colour = < 60 %.

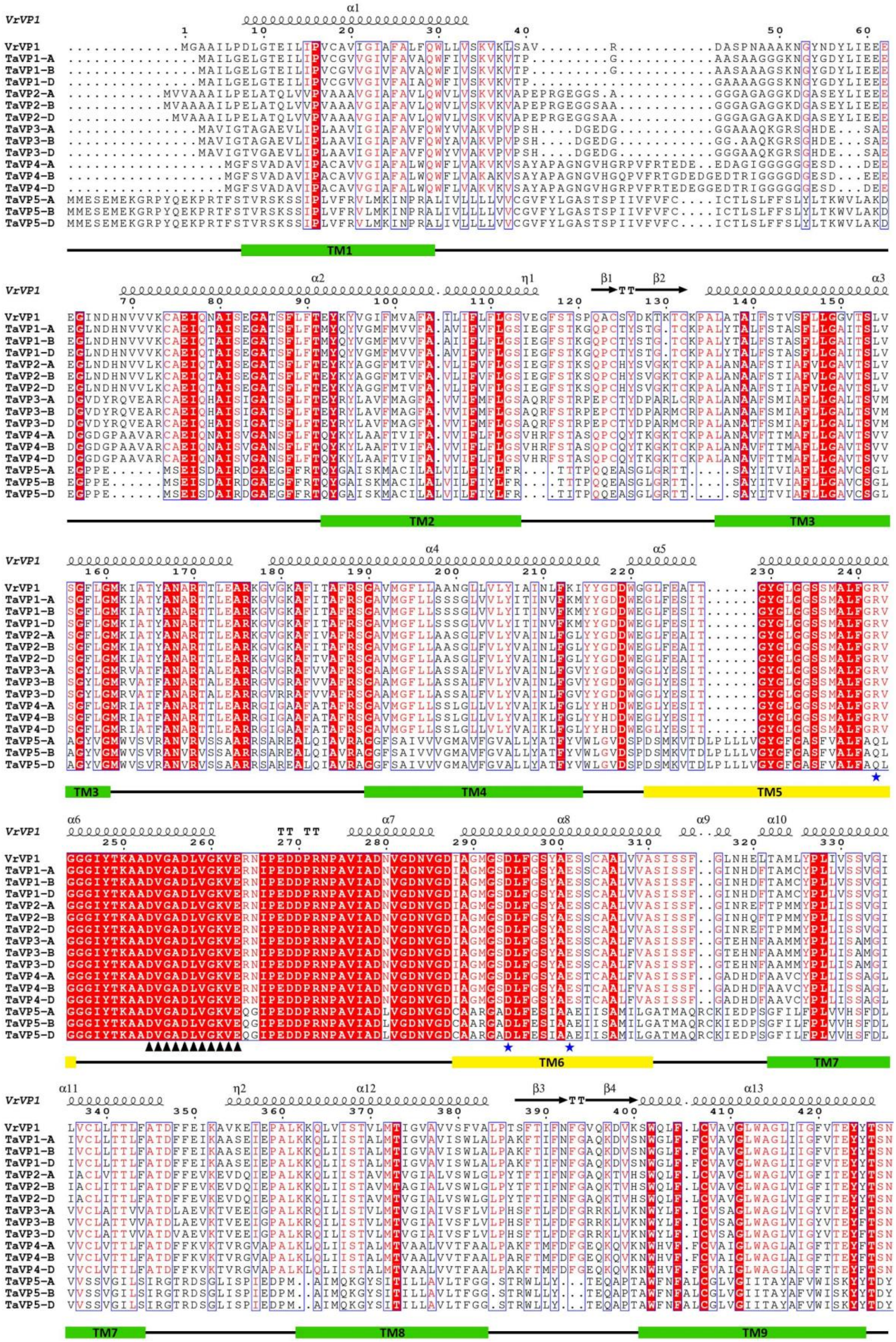
VrVP1
TaVP1-A
TaVP1-B
TaVP1-D
TaVP2-A
TaVP2-B
TaVP2-D
TaVP3-A
TaVP3-B
TaVP3-D
TaVP4-A
TaVP4-B
TaVP4-D
TaVP5-A
TaVP5-B
TaVP5-D
TM1
TM2
TM3
TM4
TM5
TM6
TM7
TM8
TM9

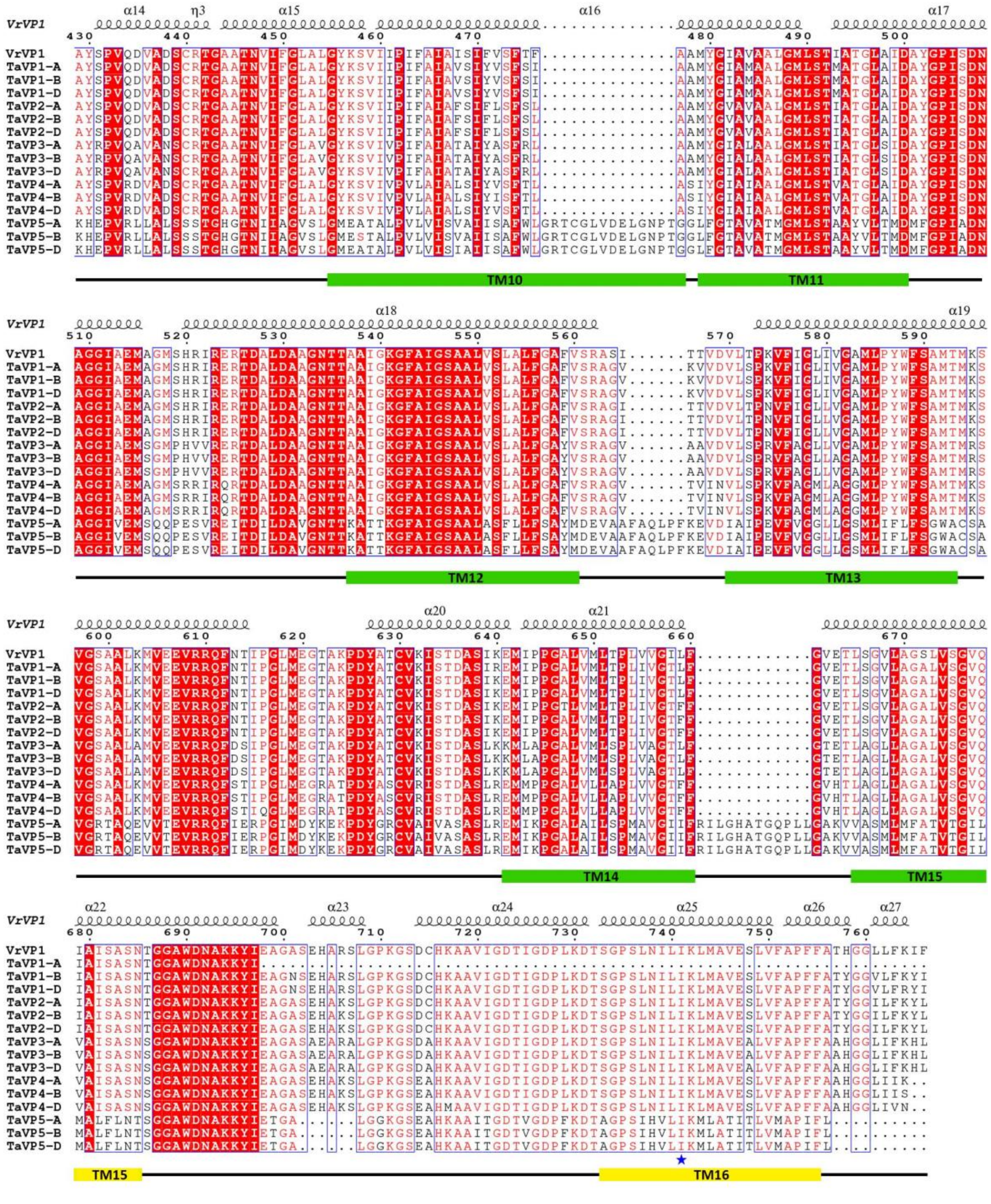

Supplementary Figure 3: **Alignment of 15 identified TaVP homeologs with *Vigna radiata* H⁺-PPase (VrVP1) protein sequence.** Red shading indicates 100 % amino acid conservation. Secondary structure elements are annotated above alignment and are based on the VrVP1 protein structure (Protein Data Bank accession 4A01). Alpha helices and beta pleated sheets are labelled with 'α' and 'β' respectively. Residues within the VrVP1 sequence known to be involved in proton translocation (stars) and pyrophosphate binding (triangles) are indicated below, as are inner (yellow) and outer (green) transmembrane domains. Alignment was created in Jalview (Waterhouse et al., 2009) using the MUSCLE algorithm (default parameters) and ESPript3.0 (Robert and Gouet, 2014) was used for annotation and visualisation of protein features.

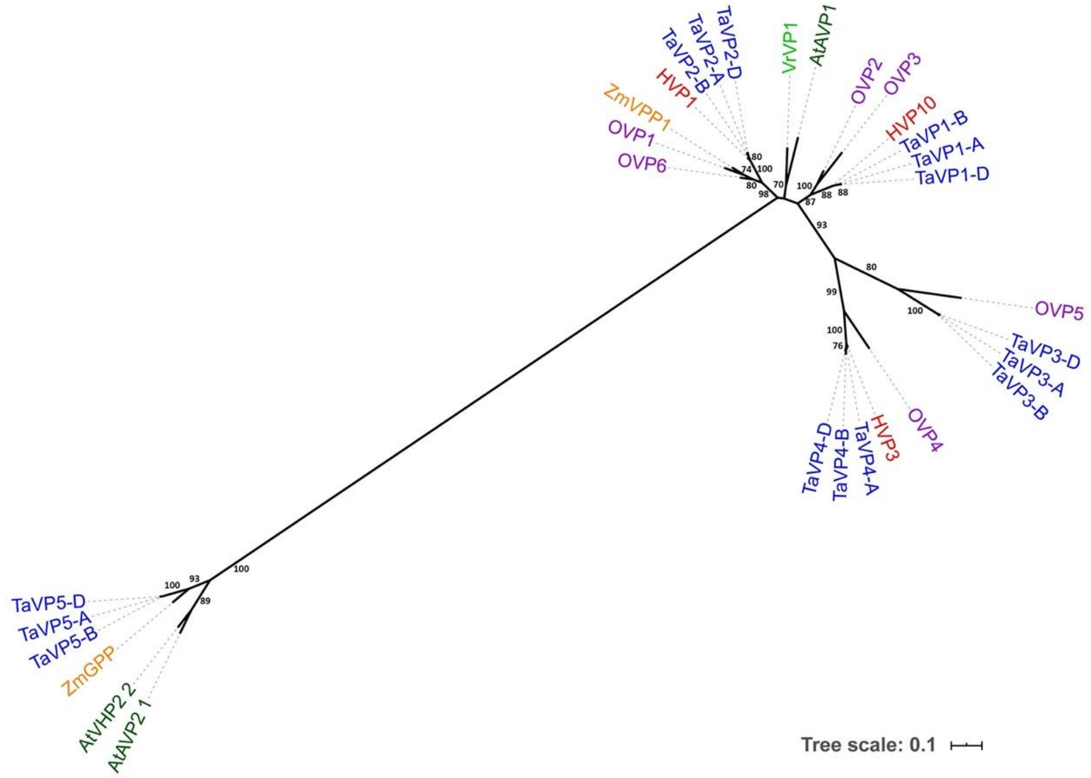

Supplementary Figure 4: Phylogenetic analysis of type I and type II H^+-PPases. Unrooted phylogenetic tree of type I and II H^+-PPase proteins from *Vigna radiata* (VrVP1), *Arabidopsis thaliana* (AVP1, AVP2, AVP3), *Hordeum vulgare* (HVP1, HVP10, HVP3), *Zea mays* (ZmVPP1, ZmGPP), *Oryza sativa* (OVP1-6) and bread wheat homeologs (TaVP1-TaVP5). Phylogeny was created in MEGA6© via the Maximum-Likelihood method and formatted with iTOL. Analysis was validated with 1000 bootstrap replicates and bootstrap support for each node is indicated. Scale bar represents 0.1 amino acid substitutions. Dotted lines are for labelling purposes only and are not included in branch lengths.

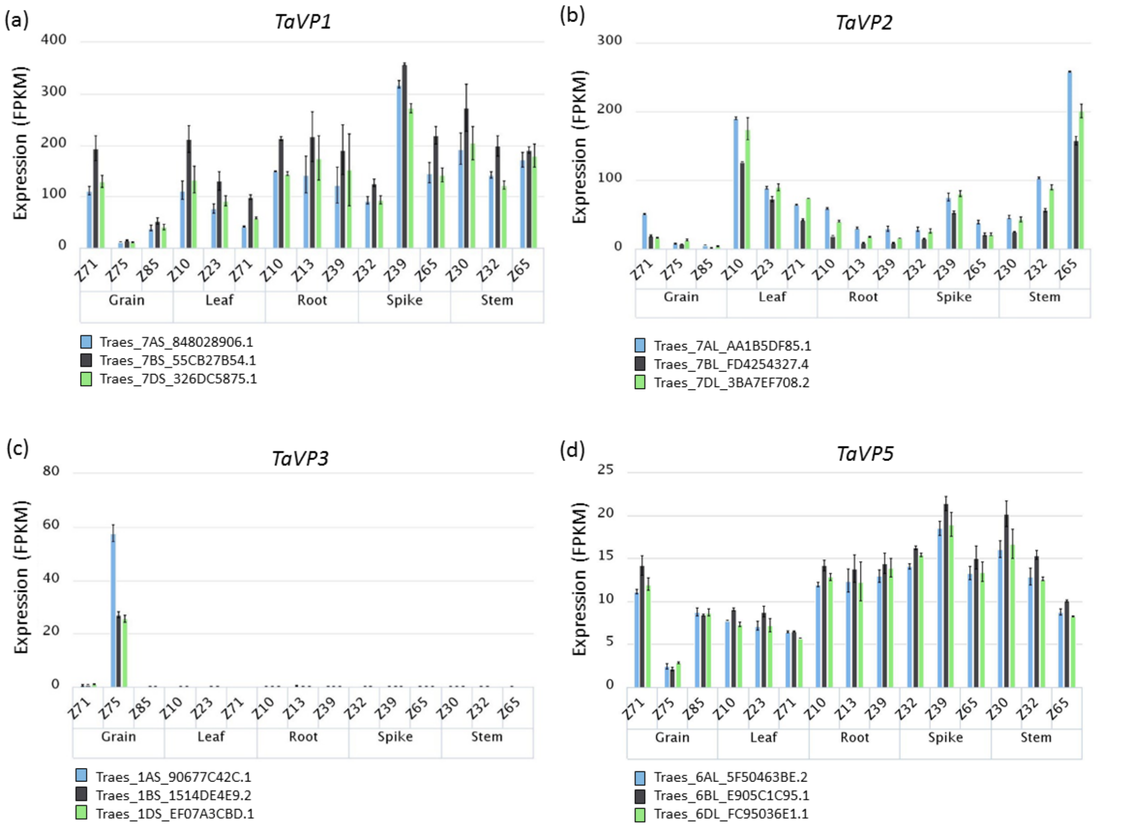

Supplementary Figure 5: RNAseq expression data of IWGSCv2 scaffolds. Each scaffold contains (A) *TaVP1* (Traes_7AS_848028906.1, Traes_7BS_55CB27B54.1, Traes_7DS_326DC5875.1), (B) *TaVP2* (Traes_7AL_AA1B5DF85.1, Traes_7BL_FD4254327.4, Traes_7DL_3BA7EF708.2), (C) *TaVP3* (Traes_1AS_90677C42C.1, Traes_1BS_1514DE4E9.2, Traes_1DS_EF07A3CBD.1), and (D) *TaVP5* (Traes_6AL_5F50463BE.2, Traes_6BL_E905C1C95.1, Traes_6DL_FC95036E1.1) genes from the A- (blue columns), B- (black columns) and D-genomes (green columns) in grain, leaf, root, spike and stem tissues under control conditions. Data was obtained from the WheatExp database (Pearce et al., 2015) and is displayed as FPKM (Fragments Per Kilobase of transcript per Million mapped reads). Values are means ± standard deviation. As *TaVP4* homeologs are missing from IWGSCv2 assembly, no RNAseq data was available in the WheatExp database.

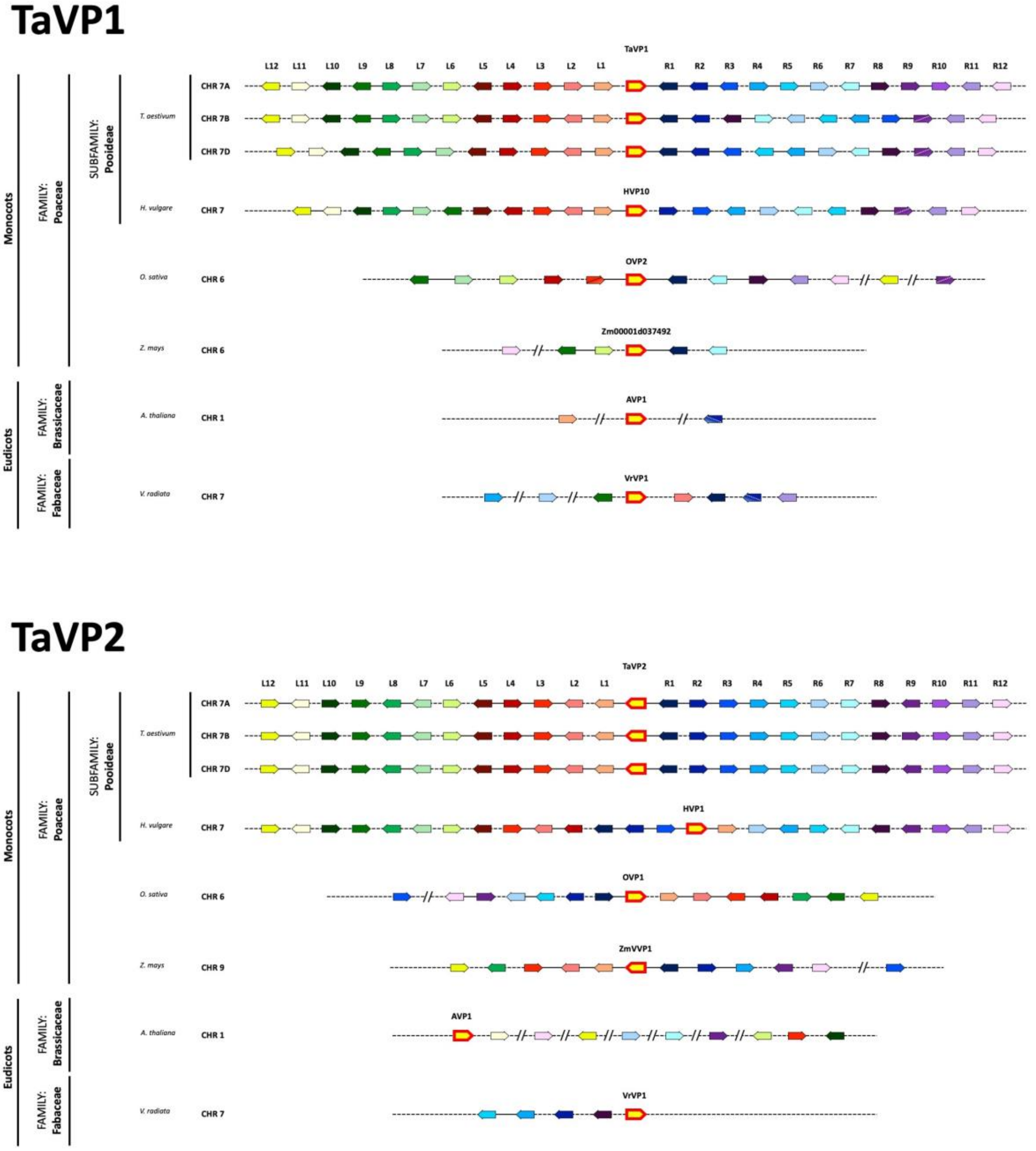

Supplementary Figure 6: Analysis of micro-collinearity of genes adjacent to TaVP1&2 across grasses and dicots. Protein sequences of the 12 upstream and downstream genes of each TaVP1&2 were obtained and their orthologs were identified by BLASTP in barley, rice, maize, Arabidopsis and mung bean. For each TaVP, orthologues are represented by arrow colour; the orientation of the genes is indicated by the direction of the arrow. Genes connected by a solid line retain their proximity, i.e. are microsyntenous, whereas those connected by a dotted line are not.

TaVP3

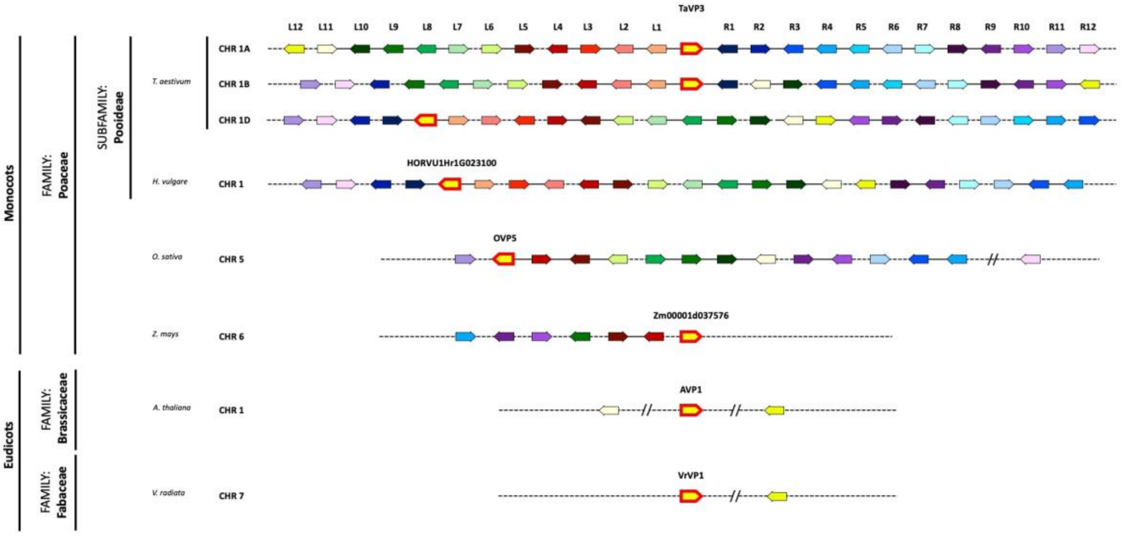

TaVP4

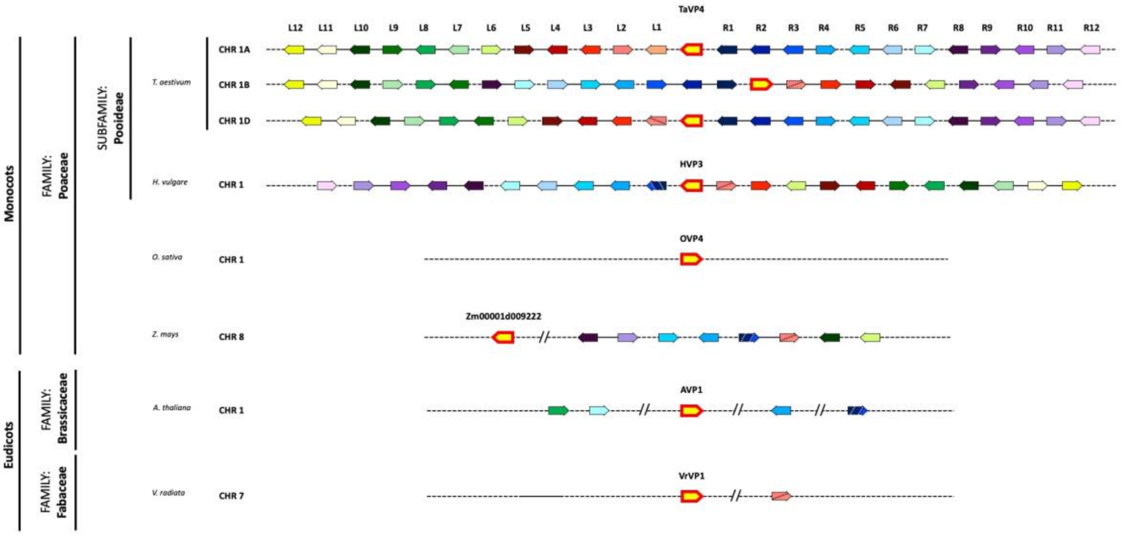

Supplementary Figure 7: Analysis of micro-collinearity of genes adjacent to TaVP3&4 across grasses and dicots. Protein sequences of the 12 upstream and downstream genes of each TaVP3&4 were obtained and their orthologs were identified by BLASTP in barley, rice, maize, Arabidopsis and mung bean. For each TaVP, orthologues are represented by arrow colour; the orientation of the genes is indicated by the direction of the arrow. Genes connected by a solid line retain their proximity, i.e. are microsyntenous, whereas those connected by a dotted line are not.

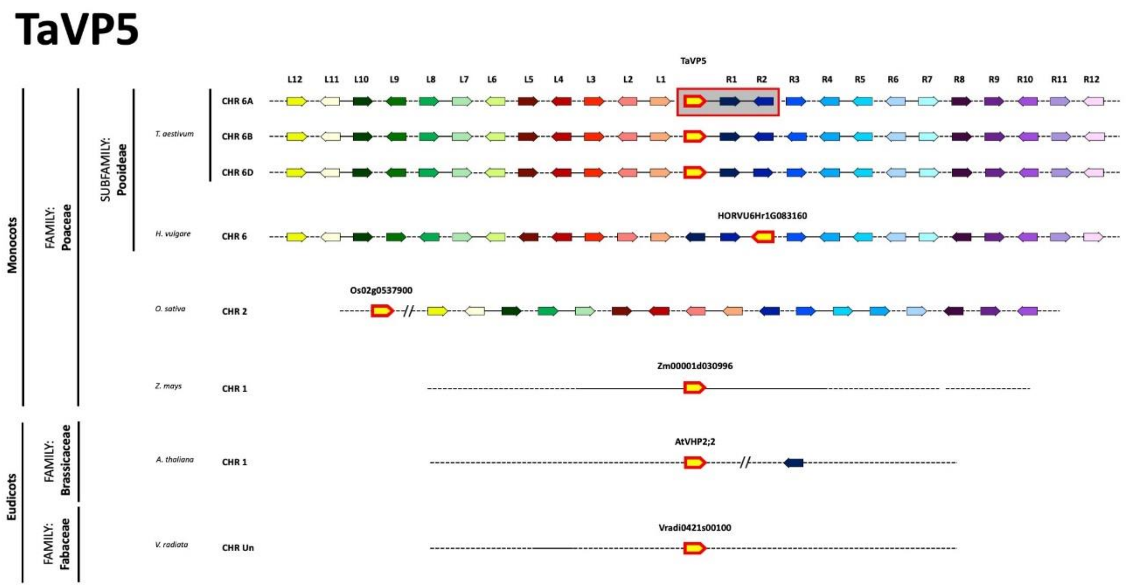

Supplementary Figure 8: Analysis of micro-collinearity of genes adjacent to TaVP5 across grasses and dicots. Protein sequences of the 12 upstream and downstream genes of each TaVP5 were obtained and their orthologs were identified by BLASTP in barley, rice, maize, Arabidopsis and mung bean. For each TaVP, orthologues are represented by arrow colour; the orientation of the genes is indicated by the direction of the arrow. Genes connected by a solid line retain their proximity, i.e. are microsyntenous, whereas those connected by a dotted line are not. For TaVP5-A the grey box indicates that in Ref1.1 one homeologue together with its two neighbours was positioned to an unallocated contig. The position and orientation of these 3 genes on Chr 6A was therefore deduced and remains to be validated.

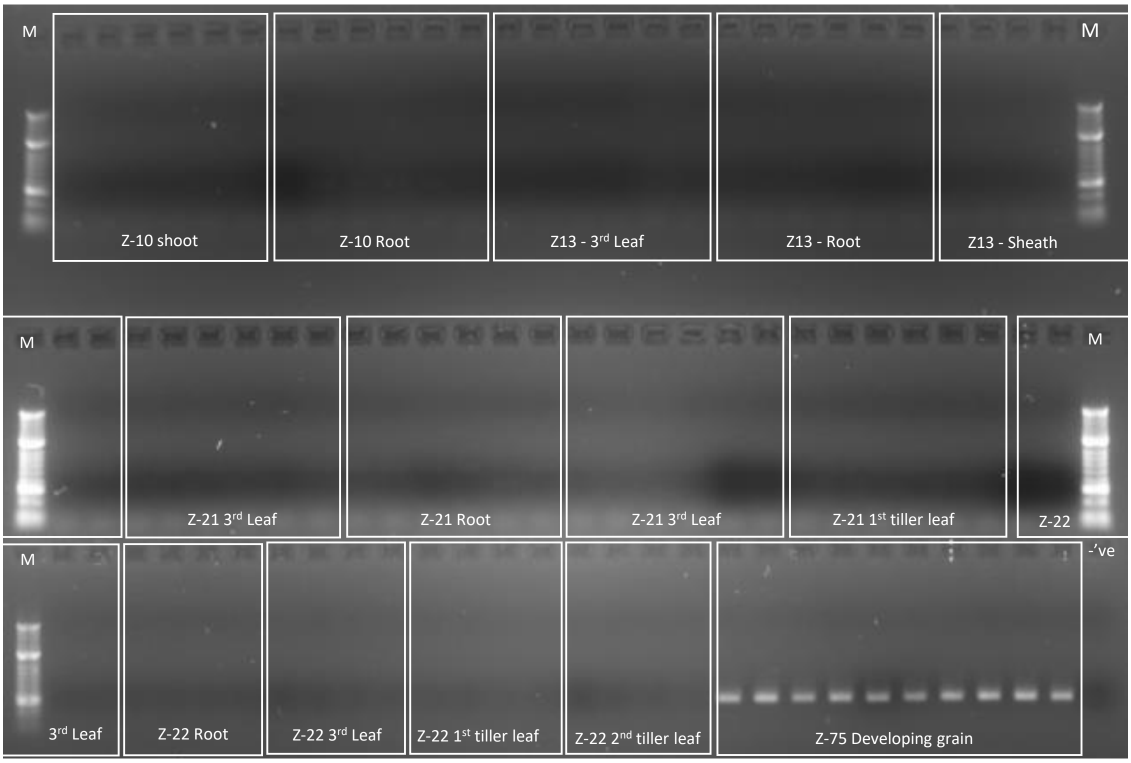

Supplementary Figure 9: Expression of *TaVP3-A* in Vigour18 cDNA samples. PCR amplification of *TaVP3-A* was performed with homeolog specific primers (Supplementary Table 1) and OneTaq polymerase (New England Biosciences, Ipswitch, United States) for 30 cycles (annealing temperature 59°C). Tissues analysed include: shoot and root at Z10; 3rd leaf, root and sheath at Z13; 3rd leaf, root, sheath and 1st leaf of the first tiller at Z21; 3rd leaf, root, sheath, 1st leaf of the first tiller and 1st leaf of the second tiller at Z22; developing grain at Z75. M = 100 bp marker (bright bands are 300 and 1000 bp). Final lane contains water as a negative control (-'ve). Figure is representative of all *TaVP3* homeologs in Vigour18, as well as the other analysed varieties.
